# PARK7/DJ-1 in microglia: implications in Parkinson’s disease and relevance as a therapeutic target

**DOI:** 10.1186/s12974-023-02776-z

**Published:** 2023-04-18

**Authors:** Frida Lind-Holm Mogensen, Andrea Scafidi, Aurélie Poli, Alessandro Michelucci

**Affiliations:** 1 Neuro-Immunology Group, Department of Cancer Research, Luxembourg Institute of Health, 6A Rue Nicolas-Ernest Barblé, L-1210 Luxembourg, Luxembourg; 2 Doctoral School of Science and Technology, University of Luxembourg, 7 Avenue Des Haut Forneuaux, L-4362 Esch-Sur-Alzette, Luxembourg

**Keywords:** Microglia, PARK7/DJ-1, Parkinson’s disease, Neurodegeneration, Neuroinflammation, Oxidative stress, NRF2, NLRP3/inflammasome, NFκB

## Abstract

Microglia are the immune effector cells of the brain playing critical roles in immune surveillance and neuroprotection in healthy conditions, while they can sustain neuroinflammatory and neurotoxic processes in neurodegenerative diseases, including Parkinson’s disease (PD). Although the precise triggers of PD remain obscure, causative genetic mutations, which aid in the identification of molecular pathways underlying the pathogenesis of idiopathic forms, represent 10% of the patients. Among the inherited forms, loss of function of *PARK7*, which encodes the protein DJ-1, results in autosomal recessive early-onset PD. Yet, although protection against oxidative stress is the most prominent task ascribed to DJ-1, the underlying mechanisms linking DJ-1 deficiency to the onset of PD are a current matter of investigation. This review provides an overview of the role of DJ-1 in neuroinflammation, with a special focus on its functions in microglia genetic programs and immunological traits. Furthermore, it discusses the relevance of targeting dysregulated pathways in microglia under DJ-1 deficiency and their importance as therapeutic targets in PD. Lastly, it addresses the prospect to consider DJ-1, detected in its oxidized form in idiopathic PD, as a biomarker and to take into account DJ-1-enhancing compounds as therapeutics dampening oxidative stress and neuroinflammation.

## Background

Microglia are the innate immune cells of the brain parenchyma playing critical roles in the communication with other glial cells and neurons. Microglia make up 5–12% of the cells in the central nervous system (CNS) and their density varies across different brain regions [1, 2]. Microglia are important for the removal of debris, injury repair, synaptic pruning and immune surveillance [3]. Their main role is to maintain the homeostasis of the inner brain milieu, acting as the first line of defense against infectious organisms, stress or other perturbations of the CNS. About 10% of genes expressed by microglia encode pattern recognition receptors (PRRs), cytokine and chemokine receptors allowing them to sense changes in the microenvironment, which is commonly referred to as the “microglia sensome” [4]. A large variety of other receptors play critical roles in microglial regulation and communication with the neuronal network as these cells are also equipped with receptors to detect other types of molecules, such as neurotransmitters and hormones [5]. During brain development, microglia support neurogenesis with neurotrophic factors, such as brain-derived neurotrophic factor, which are essential for the viability of neurons and promote synapse formation [6]. Microglial cells are also important for promoting cell proliferation and differentiation of various cell populations in the CNS [7], for instance, by stimulating the development of oligodendrocytes [8, 9]. Throughout development and during adulthood, microglia maintain the health of the neuronal environment and can even induce neuronal sprouting after injury [6, 7].

Under threatening conditions, microglia react quickly to any stimulus by undergoing morphological and functional changes, referred to as “microglia activation” represented by several stages and substates depending on the context [3, 10]. When activated, microglial cells produce cytokines, chemokines, neurotrophic and neurotoxic factors, present antigens and take on both proliferative, migratory and phagocytic properties [5]. Hence, microglia can serve both protective housekeeping functions and deleterious roles at certain stages of activation across various neurodegenerative diseases [10, 11].

PD is the second most frequent neurodegenerative disease after Alzheimer’s disease (AD) affecting 1% of the world’s population above the age of 65 and 4% of elderly people over 85 years old [12]. Furthermore, it is the fastest growing neurological disorder worldwide [13]. The progressive loss of nigrostriatal dopaminergic (DA) neurons pathologically characterizes PD. The specific loss of DA neurons in the *substantia nigra* (SN), a region of the midbrain, gives rise to the cardinal motor symptoms, including resting tremor, bradykinesia, rigidity and postural instability [14, 15]. The main neuropathological feature of PD is represented by the presence of Lewy bodies, which are proteinaceous inclusions consisting of fibrillar α-synuclein (α-syn) and ubiquitinated proteins present in the remaining nigral neurons [16]. At present, only symptomatic treatment of PD is available with long-term side effects resulting in poor patient wellbeing [17]. Hence, there is an unmet need for treating this disease.

The large majority of PD cases are idiopathic, meaning that the causes of the disease, which has a sporadic and non-familial appearance, are not known. The onset of PD is associated with environmental risk factors, such as exposure to herbicides and pesticides, brain trauma, infection and chronic stress [18–21]. However, the most important risk factor for PD is aging. In fact, epidemiological studies foresee that the prevalence of PD will increase rapidly as a larger proportion of the world´s population will become older [22]. It is currently estimated that the number of people with PD will double from 6.9 million in 2015 to 14.2 million in 2040 [13]. Several studies revealed the presence of mutations and polymorphisms related to increased risk of developing PD. Thus far, genome-wide-association studies identified 90 PD risk loci and, among them, 19 disease-causing genes have been recognized [23, 24]. Interestingly, many of them are related to genes, both autosomal dominant and recessive, whose mutations cause genetic PD, such as mutations in the gene encoding α-syn (*SNCA/PARK1*), DJ-1 (*PARK7)* and leucine-rich repeat kinase 2 (*LRRK2/PARK8*) [25–27]. 5–10% of cases are triggered by a mutation in a single gene and the focus on these specific inherited forms of PD has been a significant step for the understanding of mechanisms involved in PD [28]. The gene *PARK7* encodes a 20 kDa protein named parkinsonism-associated deglycase 1 or DJ-1, although it does not possess a protein deglycase activity [29].

This review will recapitulate the role of microglia in PD and specifically the genetic form of PD associated with the loss of DJ-1, an important protein involved in protection against oxidative stress. Recent advances have led to the discovery of a large variety of functions of DJ-1, both in the CNS [30] and peripheral immune functions [31, 32]. However, the large majority of studies have focused on DJ-1’s involvement in PD pathogenesis and the demise of dopaminergic neurons. Microglial activation, neuroinflammation and oxidative stress are hallmarks of PD, although the exact underlying mechanisms remains to be elucidated [33, 34]. Several excellent reviews have covered microglial changes in PD [35–37] and only the most important findings will be summarized herein. The study of mutations leading to PD may enable the understanding of the cellular pathways leading to neuronal protection and dampening of microglia activation in PD patients. To our knowledge, this is the first review to discuss specifically the role of DJ-1 in microglia and how it affects its genetic programs and immunological traits.

## Microglia phenotypes in Parkinson’s disease

The involvement of microglia in PD was first observed in 1988, with the demonstration that activated microglia in the SN of patients displayed high expression levels of MHC class II, an important molecule for antigen presentation [38]. Later, the existence of activated microglia in SN post-mortem tissue from PD patients has been confirmed [39, 40]. Furthermore, in patients with other Parkinsonian syndromes, degenerating neurons in the SN are closely associated with large numbers of activated microglia [41, 42]. However, activated microglia have not only been detected in the SN, but also in the putamen, hippocampus, as well as transentorhinal cingulate and temporal cortex, where they express the lysosomal activity marker CD68, the scavenger receptor toll-like receptor 2 (TLR2), the intracellular adhesion molecule one (ICAM-1) and the integrin receptor CD11a [41, 43–47]. Various cytokines have also been detected, which further points to an activated or deregulated immune response in these patients. For example, tumor necrosis factor (TNF), transforming growth factor (TGF)-α, TGF-1β, IL-1β, IL-2 and IL-6 were elevated at the protein level in the striatum and cerebrospinal fluid (CSF) [48–52]. A recent study found that α-syn-accumulating microglial cells develop a strong reactive state with an excessive production of reactive oxygen species (ROS) and pro-inflammatory cytokines in an in vivo mouse model of lentiviral-mediated selective α-syn accumulation in microglia. This model showed DA neuronal degeneration due to reactive microglia and interferon γ (IFN-γ) secreting immune cells infiltrating the brain [53]. Of note, microglia can distribute α-syn among them via tunneling nanotubes to share its load, a process that is impaired in cells carrying the genetic PD mutation LRRK2 G2019S, increasing neuroinflammation and the death of neurons [54].

Microglial activation and neuroinflammation in PD support the degeneration of the DA neurons in the nigrostriatal pathway, but exactly how and when it occurs is not yet known [39, 40, 55]. The selective loss of neurons in the SN may be explained by a lower antioxidant ability of DA neurons, rendering them more susceptible to both oxidative stress and inflammatory insults, partly mediated by microglia [56–58]. Additionally, DA neurons in the SN are particularly exposed to oxidative stress as the dopamine metabolism can give rise to various endogenous toxins if not properly removed [56, 59]. Furthermore, the density of microglial cells is considerably higher in the SN compared to other brain regions, which is an additional factor that might explain the susceptibility of these neurons [1, 2, 60]. In line with this observation, midbrain microglial cells show an upregulation of inflammatory response genes compared to the corresponding cells in the striatum [61]. In this context, it is relevant to switch the focus from a DA neuron-centered toward a neuroinflammation-contributing disease, thereby setting microglial cells as crucial players in PD development and progression [53, 62].

## Roles of PARK7/DJ-1 and its involvement in Parkinson’s disease

**Fig. 1:**
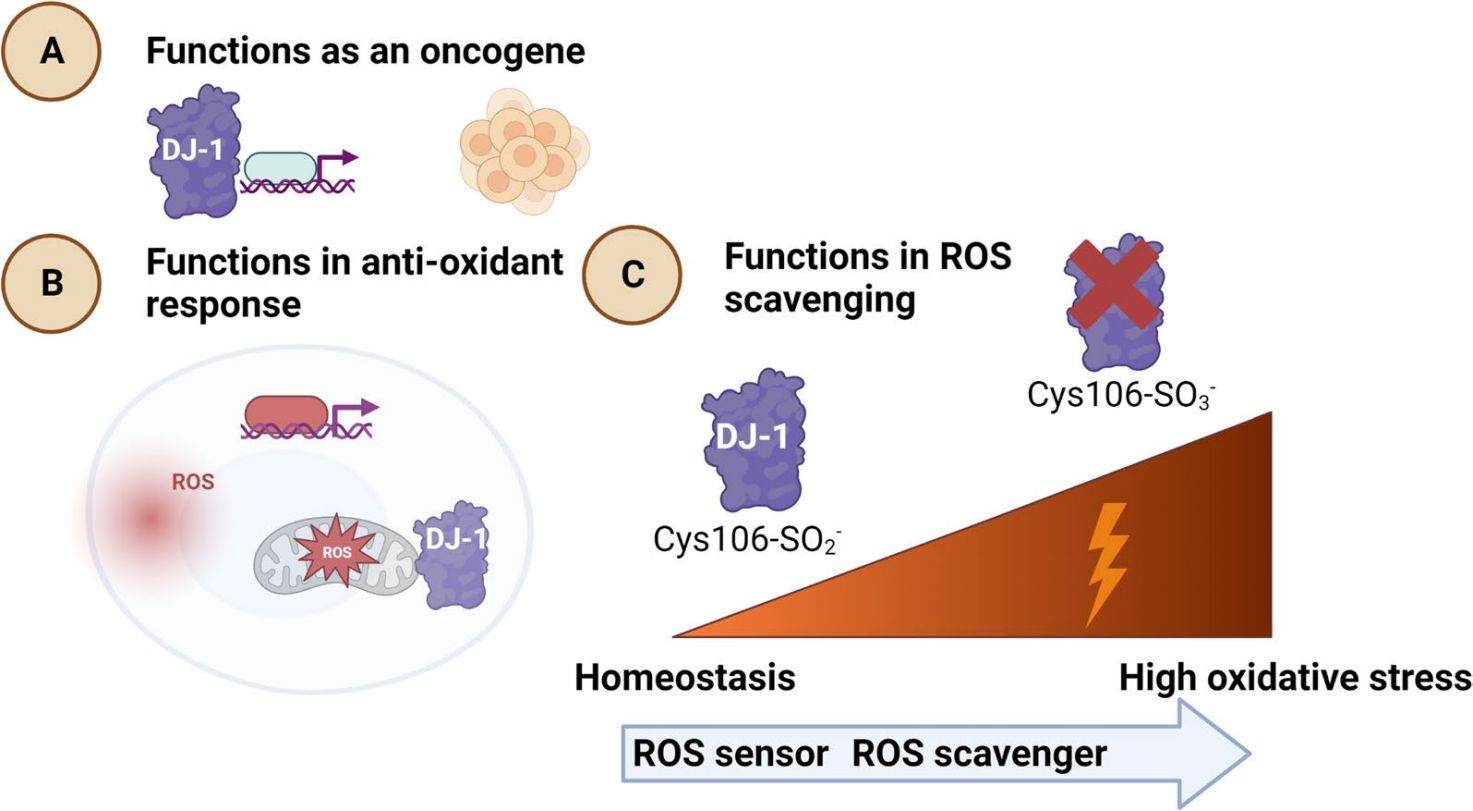
Functions of DJ-1. DJ-1 has a plethora of functions and was originally described as an oncogene **A** due to its involvement in cellular transformation and transcriptional regulation. **B** DJ-1 protects against oxidative stress both indirectly by stabilizing transcription factors inducing an antioxidant response or **C** directly by scavenging reactive oxygen species (ROS) both in the mitochondria and cytoplasm and indirectly by stabilizing transcription factors inducing an antioxidant response. The cysteine residue on position 106 (Cys106) within the DJ-1 protein acts as a sensor of redox state and can be oxidized to sulfinate (-SO_2_^−^) and further to sulfonate (-SO_3_^−^). Cys106-SO^2−^ has cytoprotective functions, whereas excessive oxidative stress can induce its overoxidation Cys106-SO_3_^−^ and thereby its loss of function. Figure created with www.BioRender

The expression of DJ-1 is especially high in tissues with a high metabolic rate, such as the liver, kidney and CNS. In the brain, DJ-1 is mainly expressed by microglia, astrocytes and neurons [63, 64]. Although DJ-1 is predominantly found in the cytosol, it is also associated with the nucleus and mitochondria [64–66]. The gene was first described in 1997 as an oncogene with transforming properties affecting ras-dependent transformation **(**Fig. 1A**)** [66]. Its loss of function was later defined in 2001 as a causative factor for a familial form of early-onset PD with an autosomal recessive inheritance (Box 1) [67, 68]. The DJ-1 protein is involved in several cellular activities [69]. Among others, it regulates mitochondrial functions, acts as a protease, a RNA-binding protein and a chaperone delivering proteins to the proteasome [70], regulates transcription and is involved in autophagy [71] (Table 1). However, the most prominent function of DJ-1 is to protect cells against ROS [72] (Fig. 1B). ROS are chemical reactive species containing oxygen (e.g., peroxides, hydroxyl radicals and superoxides) induced by UV radiation, tobacco smoke, drugs, xenobiotics and pesticides, the latter two being of high relevance for the development of PD [73]. Notably, a recent study investigating the possible inhibitory effects of one hundred pesticides on DJ-1 uncovered that 15 of them were effective inhibitors of the human DJ-1 protein [74]. All of them were previously linked to PD, such as paraquat, which causes oxidative stress, DA neuronal loss and PD symptoms (Table 1) [75, 76]. Hence, the inhibition of DJ-1 by pesticides might represent a biological effect contributing to ROS accumulation and the development of PD. DJ-1 also possesses metal-binding properties, which are lost when DJ-1 is mutated, thus underlining the importance of DJ-1 to protecting against the toxicity of heavy metals, such as copper and mercury [77], since the chronic exposure to these metals is linked to PD and contributes to its progression inducing neuronal loss through neuroinflammation, oxidative stress, DNA damage, mitochondrial dysfunction, and apoptosis [78]. Not surprisingly, the combination of various environmental toxicants throughout life, confers even a greater risk of developing PD [79]. Further research is warranted to delineate by which mechanisms different pesticides, heavy metals and probably yet unknown toxicants [80, 81] increase ROS accumulation and the susceptibility to PD.

**Table 1:** DJ-1 functions described to date

Function	Model	References
Functions as an oncogene		
Suppresses phosphatase PTEN	*Drosophila melanogaster*, COS7 (monkey fibroblast cell line), NIH3T3 (mouse fibroblast cell line)	[177]
Alters p53 signaling	HEK293T (human kidney cell line), A549 (human lung adenocarcinoma cell line), H1299 (human lung carcinoma cell line), DJ1 KO primary mouse fibroblast	[178]
	N2a (mouse neuroblastoma cell line), HEK293T, A549, H1299, *Danio rerio*	[179]
	HEK293T, H1299, HeLa (human cervical cancer cell line),	[180]
	ME180 (mouse tumor associated endothelial cell line)	[181]
Cooperative transforming activity with H-*ras*	NIH3T3, HeLa, Cos I (monkey fibroblast cell line), 3Y1 (rat fibroblast cell line)	[66]
Functions in antioxidant responses		
Upregulates glutathione (GSH) synthesis Via the inhibition of GSH inhibitor protein glutathione-specific gamma-glutamylcyclotransferase (CHAC1)	N27 (rat dopaminergic neuron cell line), primary rat mesencephalic cells	[97]
	HEK293T, primary mouse astrocytes	[98]
Stabilizes NRF2 Via the upregulation of thioredoxin 1	Primary mouse embryonic fibroblasts, Huh7 (human liver cancer cell line), H157 (human oral squamous carcinoma cell line)	[90]
	SH-SY5Y (human dopaminergic neuroblastoma cell line), HeLa	[118]
Protects against oxidative stress to prevent cell death Via an interaction with Daxx to suppress cell death Via the binding to subunit of NADPH oxidase to prevent oxidative burst DJ-1 protects against cell death induced by oxidative, but not nonoxidative, stress following MPTP-treatment in vivo Stronger intracellular ROS response with and without LPS treatment in DJ-1 deficient microglia compared to wildtype	H1299, HEK293T	[96]
	NIH3T3, SH-SY5Y	[92]
	HeLa, SH-SY5Y	[182]
	SH-SY5Y	[72]
	Bone marrow-derived macrophages from mice and humans, THP-1 (human monocytic cell line), RAW 264.7 (mouse monocytic cell line)	[113] [112]
	MPTP-model of PD in DJ-1 KO mice	[110]
	DJ-1 deficient N9 (immortalized murine microglia cell line)	[111]
Other functions of DJ-1		
Interacts with PINK1 and PARKIN	SH-SY5Y, HEK293 SH-SY5Y	[183, 184]
Acts as a redox sensitive chaperone	SH-SY5Y, HEK293, Undifferentiated murine ES cells, HeLa, CAD (mouse neuroblastoma cell line)	[70]
Prevents damage to proteins and metabolites caused by 1,3-biphosphoglycerate	HCT 116 (human colon cancer cell line)*, Drosophila melanogaster, Schizosaccharomyces pombe, Escherichia coli*	[185]
Autophagy and phagocytosis of α-syn and mitochondria	N9 (mouse microglia cell line), primary mouse microglia	[71]
	DJ-1 KO mouse embryonic fibroblasts, DJ-1 KO mouse primary cortical neurons, DJ-1 KO mouse primary lymphoblasts, H1299	[192]
	DJ-1 KO mouse embryonic fibroblasts, primary human fibroblast from DJ-1 mutated patients (E46D homozygous mutation)	[186]
Mitochondrial homeostasis	DJ-1 KO mouse embryonic fibroblasts, DJ-1 KO mouse primary cortical neurons, DJ-1 KO mouse primary lymphoblasts, H1299	[187]
	N9 (mouse microglia cell line), primary mouse microglia	[71]
	SN4741 (mouse dopaminergic neuron cell line)	[188]
Controls metabolic pathways Promote pyruvate dehydrogenase (PDH) activity by binding to PDHE1-β, a component of the PDH complex, inhibiting phosphorylation and thereby promoting oxidative phosphorylation	*Drosophila melanogaster*, DJ-1 deficient SH-SY5Y	[189]
	Aged DJ-1 KO mice	[32]
Acts as a protease	H1299, HEK293, HEK293T	[96]
	DJ-1 crystallography (in silico prediction), (Flp-In)NIH3T3	[99]
	D2 (mouse mammary gland tumor cell line), HEK293T	[190]
Modulates androgen-receptor signaling	HEK293T, TM4 (mouse Sertoli cell line), HepG2 (human liver carcinoma cell line), Cos I, CV1 (monkey fibroblast cell line)	[191]
	COS7	[192]
	H1299, HEK293T, LNCaP (human prostate cancer cell line), LAPC4 (human prostate cancer cell line)	[193]
Alters dopamine receptor signaling	Acute striatal slices from DJ-1 KO mice	[194]
	HEK293T	[195]
	SH-SY5Y, SK-N-SH (human neuroblastoma cell line)	[196]
Inhibits aggregation of α-synuclein	SH-SY5Y, DJ-1 KO mice	[197]
	HEK293T, H4 (Human neuroglioma cell line), undifferentiated murine ES cells, HeLa, SH-SY5Y	[198]
	Undifferentiated murine ES cells, HeLa, CAD	[70]
Binding of STAT1 phosphatase	Primary mouse DJ-1 KO microglia and astrocytes, DJ-1 KO mouse cortical slices, BV-2 (mouse microglia cell line)	[131]
Binding of p65 (subunit of NFκB)	DJ-1 KO mouse, BV-2	[133]

At a late stage of the disease, the SN of PD patients exhibits typical characteristics associated with ROS accumulation, including decreased glutathione (GSH) levels together with increased lipid peroxidation and manganese superoxide dismutase activity and upregulated oxidative stress biomarkers [59, 82]. A small amount of ROS is produced under homeostatic conditions in the complex IV of the mitochondria during oxidative phosphorylation and plays an important role in cell signaling [83]. However, macrophages and microglia can substantially increase the production of ROS when exposed to pathogen-associated molecular patterns (PAMPs), neurotoxins and various inflammatory peptides [84, 85]. As a result, they work as bactericidal mediators getting rid of pathogens via for example the nicotinamide adenine dinucleotide phosphate (NADPH) oxidase-mediated release of ROS, also called oxidative burst [86]. NADPH oxidase is a membrane-bound enzyme complex producing ROS by catalyzing the production of superoxide from oxygen [36]. Interestingly, the catalytic subunit of the NADPH oxidase is upregulated in PD [87]. It was demonstrated that when activated via lipopolysaccharide (LPS), primary microglia from rats produced neurotoxic extracellular ROS, via NADPH oxidase, leading to blood–brain barrier (BBB) dysfunction in vitro [88]. Moreover, NADPH oxidase in microglia primarily produced neurotoxic extracellular ROS. Thus, it is indirectly demonstrated that NADPH-mediated ROS production by microglia can contributes to neurotoxicity [62]. Of relevance for the PD pathophysiology, α-syn uptake by microglia leads to ROS production via NADPH oxidase [89].

DJ-1 can protect cells against ROS both directly by scavenging ROS and indirectly by stabilizing nuclear factor erythroid factor-E2 related factor 2 (NRF2), which is a master regulator of antioxidant response genes [90] (Fig. 1B). Under oxidizing conditions, DJ-1 can self-oxidize by forming cysteine-sulfinic acid [91]. The cysteine residue on position 106 (Cys106) of DJ-1 is oxidized when cells are exposed to oxidative stress. Cys106 is critical for the biological function of DJ-1, with its oxidized form Cys-SO_2_H representing the active form [92], while further oxidation of DJ-1 to Cys-SO_3_H leads to its loss of function (Fig. 1C) [93]. Furthermore, oxidative stress stimulates the degree of translocation of DJ-1 into the mitochondria [91, 94]. Under homeostatic conditions, DJ-1 binds to subunits of the mitochondrial complex I regulating its activity [95]. However, the binding of DJ-1 to complex I subunits are enhanced under oxidative stress. Mitochondria-targeted sequence-conjugated DJ-1 is more protective against cell death induced by oxidative stress than when in the cytoplasm [94]. Without a proper DJ-1 function, a decreased antioxidant response will occur in the affected cells [96]. Another mechanism by which DJ-1 limits cellular ROS accumulation is via the upregulation of GSH levels through an increase of the rate-limiting enzyme glutamate cysteine ligase [97]. A recent study revealed further mechanistic insights, as DJ-1 was shown to regulate GSH levels in astrocytes via its binding to the glutathione-specific gamma-glutamylcyclotransferase 1 (CHAC1), a protein involved in GSH degradation (Table 1) [98].

Interestingly, a PD-related mutation in DJ-1, the L166P mutant, blocks the ability of DJ-1 to dimerize and the monomer form of DJ-1 is rapidly degraded [99–101]. Therefore, L166P mutants have a diminished protection against oxidative stress due to lower DJ-1 concentrations [97]. Patients with a homozygous DJ-1 deletion show decreased [^18^F]-fluoro-L-3,4-dihydroxyphenylalanine uptake and cerebellar hypometabolism, which is comparable with alterations seen in idiopathic PD (iPD) patients (Box 1) [67, 102, 103]. Only one post-mortem study of a DJ-1 patient has been characterized so far and the neuropathological analysis showed significant DA neuronal loss and Lewy bodies with α-syn aggregates in various brain regions as typically described in post-mortem studies of iPD patients [104, 105]. Significant gliosis was also described in this post-mortem study, which is also similar in iPD post-mortem studies and is in line with the notion that microglia are highly activated by α-syn leading to secretion of neurotoxic substances, including TNF and ROS [105].

The abovementioned studies mostly investigated DJ-1 functions using neurons or cancer cell lines, as listed in Table 1 and reviewed in [106–109]. Comparatively, mechanistic insights specifically addressing microglia are discussed in the next section.

Box 1. Age of onset and phases of Parkinson’s disease Parkinson’s disease (PD) is an incredibly complex disease, which is probably composed of many diseases collectively referred to as PD. One can distinguish PD in terms of its age of onset; young-onset PD (YOPD) is diagnosed between 21 and 40 years of age [207]. Approximately 3–5% of cases of PD worldwide starts at this young age. YOPD can be further subdivided into rare juvenile parkinsonism, which starts before the age of 21 and the YOPD where the onset is between 21 and 40 years of age. YOPD can be distinguished from late-onset PD (LOPD) (diagnosis after 40 years of age), as it often has a genetic etiology and clinically the patients present with dystonia and levodopa-induced dyskinesia [207]. LOPD on the other hand, is often idiopathic and in the Western world the mean age of PD onset is in the 6th decade of life [13]. There are three phases of PD; (1) The pre-clinical phase, where the degeneration of DA neurons has already begun, but the patient has no symptoms; (2) The prodromal phase where symptoms are present, such as hyposmia, depression and/or constipation, but the patient does not live up to the diagnostic criteria (in addition to bradykinesia, the patient should show one or more motor symptoms, such as tremor at rest, stiffness or rigidity of arms or legs) [208]; (3) The clinical phase where symptoms are clearly recognizable and manifested.

## Functions of DJ-1 in microglia

### DJ-1 functions in antioxidant responses

**Fig. 2:**
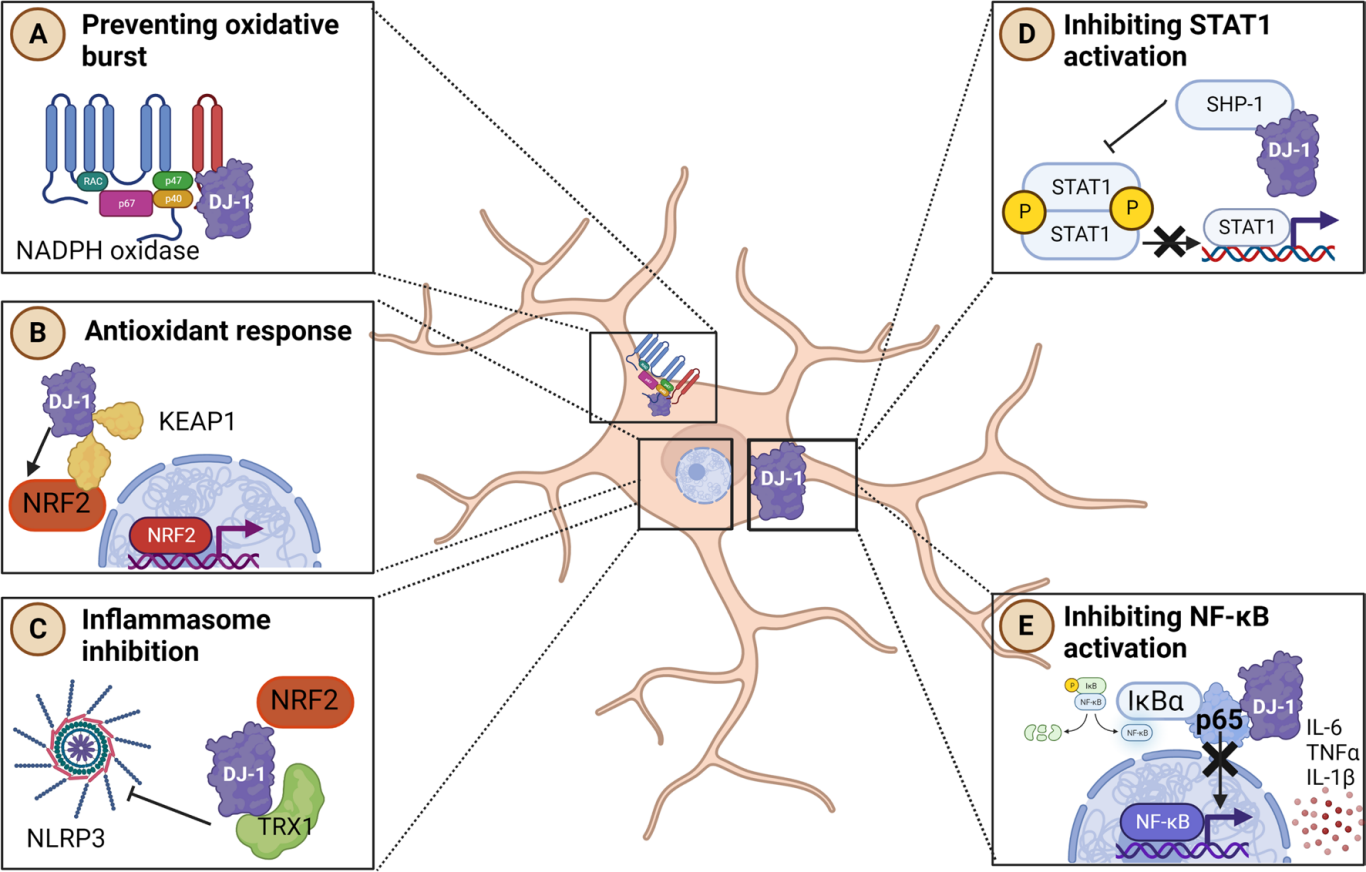
Functions of DJ-1 in microglia. **A** DJ-1 binds the p47 (green) unit of the NADPH oxidase preventing oxidative burst. **B** By preventing the binding of KEAP1 (yellow) to NRF2 (red), DJ-1 activates NRF2, which acts as a transcription factor regulating anti-oxidative responses. **C** DJ-1–NRF2 axis inhibits the inflammasome activation via TRX1. **D** DJ-1 facilitates the interaction of SHP-1 with STAT1, thereby inhibiting the STAT1 pathway. **E** DJ-1 binds the p65 subunit of the NFκB, thereby inhibiting the activation of the NFκB pathway. *NRF2* NF-E2 related factor 2, *KEAP 1* Kelch-like ECH protein 1, *TRX1*  Thioredoxin-1, *SHP-1* Src-homology 2-domain containing protein tyrosine phosphatase-1, *STAT-1* Signal transducer and activator of transcription 1, *IKbα* NF-κB inhibitor α, *NFκB* nuclear factor kappa light chain enhancer of B cells. Figure created with www.BioRender

The balance between the production of oxidative species and their removal determines the oxidative stress of a cell or a tissue. One of the many functions of DJ-1 is to protect cells against oxidative stress-induced cell death [96, 110]. DJ-1-deficient N9, an immortalized microglia cell line, showed higher intracellular ROS levels compared to control microglia both with and without LPS stimuli (Box 2) [111]. Similarly, nitric oxide (NO) levels were higher in DJ-1-deficient microglia, both at baseline and after 16-h treatment with LPS [111]. Two independent studies showed that DJ-1 interacts with a subunit of NADPH oxidase in macrophages [112, 113]. Specifically, in bone marrow-derived macrophages (BMDMs) from mice and humans, DJ-1 can bind p47phox, which is a critical component of the NADPH oxidase complex (Fig. 2A). The binding of DJ-1 to p47phox disrupts the NADPH oxidase complex facilitating its ubiquitination and degradation, ultimately resulting in decreased ROS production [112]. A study conducted in a sepsis model showing that enhanced bactericidal responses in DJ-1 KO mice are associated with decreased mortality compared to wildtype mice, which underline the importance of the DJ-1–ROS axis in response to life-threatening infections (Box 3) [113].

DJ-1 deficiency in macrophages and microglia does not only lead to increased ROS production through the NADPH oxidase complex, but their antioxidant response is also impaired. Specifically, DJ-1 deficient microglia exhibit changes in GSH levels. Both the glutamine and serine pathways are important for de novo synthesis of GSH as they provide the precursors glutamate, serine and glycine [114]. The loss of DJ-1 in neurons and microglia modifies the central metabolism by decreasing the glutamine influx and the biosynthesis of serine [114]. Decreased levels of GSH in DJ-1 deficient cells and a weak constitutive activation, characterized by an increase in TNF, cis-aconitate decarboxylase 1 and iNOS compared to wildtype (WT) microglia have been detected [114]. Interestingly, a recent paper found significantly higher GSH levels in the medial prefrontal cortex of DJ-1 KO mice, which likely represents a compensatory mechanism to overcome the loss of DJ-1 and thereby the loss of its anti-oxidative mechanisms [115]. Multiple studies have been carried out in DJ-1 KO mice to delineate DJ-1 function (Box 3). DJ-1 deficient mice show subtle motor- and cognitive symptoms with old age and when treating DJ-1 KO mice with the neurotoxin 1-methyl-4-phenyl-1,2,3,6-tetrahydropyridine (MPTP), a greater loss of DA neurons occurred compared to WT mice, thus suggesting that DJ-1-deficient mice are more vulnerable to neurotoxins [110] (Table 1).

Box 2. Lipopolysaccharide (LPS) as a molecule activating microglia and inducing neurotoxicity LPS is a component of the Gram-negative bacteria cell wall able to stimulate a strong immune activation both in vitro and in vivo. LPS is used to model sepsis if injected in the bloodstream, while it recapitulates neurodegenerative processes when injected directly in the brain or at high doses in the periphery. Briefly, LPS interacts with toll-like receptor 4 (TLR-4) and induces the activation of the transcription factor nuclear factor kappa-light-chain enhancer of activated B cells (NFκB) resulting in transcription of pro-inflammatory mediators, including IL-6 and IL-1β, leading to an acute inflammatory reaction in both mice and human microglia and macrophages [209, 210]. It is widely established that microglia, as the first line of defense in the CNS, produce inflammatory cytokines and excessive ROS after a LPS challenge, but the physiological relevance of LPS-induced microglia activation to model neurodegenerative diseases can be questioned and should be interpreted with caution. However, LPS-induced neurotoxicity is strictly dependent on microglia, strongly suggesting that microglia initiate the damage to neurons [62, 211, 212]. Both in vivo and in vitro studies show the progressive and accelerating loss of DA neurons over time. When LPS is systemically injected, microglia are activated via TNF and mediate neurodegeneration specifically of DA neurons in the SN [213]. Prenatal exposure to LPS can lead to the loss of DA neurons and microglia activation in adulthood [214–216].

Box 3. Studies of DJ-1 KO mouse models to delineate DJ-1 functions In order to study the functions of DJ-1 in vivo and to reveal how DJ-1 is associated with PD, researchers have created a DJ-1 knockout (KO) mouse model [177, 217] (Table 1). Albeit no significant DA neuronal loss was detected in the DJ-1 KO mouse model, a study testing a battery of behavioral paradigms to elucidate both motor and dopaminergic functions in DJ-1-deficient mice from 2 to 24 months of age, showed gait abnormalities in young mice, which progressed with aging [194, 217–219]. Hypokinesia was seen in 5- to 24-month-old DJ-1 KO mice together with a progressive decrease in grip strength. However, this was not associated with changes in dopamine levels, tyrosine-hydroxylase (TH) positive DA neurons or D2 auto receptor sensitivity [220]. Similar findings were also described in 18- to 27-month-old DJ-1 KO mice [221]. Additionally, oxidative damage and α-syn inclusions were not found in DJ-1 KO mice across their lifespan [221]. Only a subset of highly backcrossed DJ-1 KO mice showed DA neuronal loss [222]. However, the knockout of DJ-1 in rats results in progressive neuronal loss characterized by motor deficits, gait dysfunctions and up to 50% neuronal loss in 8-month-old rats [223]. When knocking out other PD-related genes in mice, such as PTEN-induced putative kinase (*PINK1*) and parkin, an E3 ubiquitin ligase *(PARK2)*, there were not significant DA neuronal loss [194, 218, 224]. All of these observations indicate that defects, deficiencies or complete knock out of PD-related genes, including *PARK7*/DJ-1, alone are not sufficient for the manifestation of typical PD symptoms in mice. However, even though these models do not recapitulate late-stage PD pathology, they represent valuable paradigms to study its onset and prodromal phases in relation to the specific pathogenesis (Box 1).

### The interplay between DJ-1, NRF2 and NLRP3

DJ-1 prevents the binding of NRF2 to Kelch-like ECH Associated Protein 1 (KEAP1), a protein important for the ubiquitination of NRF2 [90]. NRF2 is an essential regulator of the endogenous anti-oxidative response in the cell as it induces key genes involved in the antioxidant response [90]. Thus, by preventing the binding of KEAP1 to NRF2, DJ-1 stabilizes NRF2 and increases its expression levels without a direct binding of DJ-1 to it (Fig. 2B) [116]. The interplay between DJ-1 and NRF2 also acts as a suppressor of the inflammatory inducer nod like receptor pyrin domain containing 3 (NLRP3) [116]. Hence, NRF2 suppresses NLRP3 inflammasome activity via thioredoxin 1 (TRX1) [117] and DJ-1 was shown to upregulate TRX1 expression [118]. Interestingly, a recent study showed that NLRP3 is negatively regulated by DJ-1 in a murine microglia cell line (BV-2) [116] (Fig. 2C). NLRP3 is an inflammatory mediator in microglia that acts as an intracellular sensor of stress and can induce the release of inflammatory cytokines, such as IL-1β and IL-18 [119, 120] (Fig. 2C). Importantly, the CNS is especially sensitive to these cytokines as various neural cell types express receptors for IL-1β and IL-18 [121]. In PD-related studies, treatment of cultured microglia and neurons with α-syn activates NLRP3 inflammasome and so the subsequent IL-1β release [122–124]. NLRP3 amplifies neuroinflammation via astrogliosis and microgliosis, which exacerbate demyelination in a mouse model of neurodegeneration [125]. Furthermore, NLRP3 represents one of the most common inflammatory pathways implicated in PD [126, 127]. Indeed, NLRP3 supports the progression of PD in mouse and rat models [122, 128, 129].

### Interaction of DJ-1 with the JAK–STAT, interferon and NFκB pathways

The Janus kinase/signal transducer and activator of transcription (JAK/STAT) signaling pathway initiates secretion of many cytokines resulting in cell growth and death playing critical roles in neuroinflammatory processes associated to neurodegenerative diseases [130]. Among the various signaling pathways, DJ-1 interacts with the JAK–STAT pathway in microglia [131]. Specifically, DJ-1 facilitates the interaction of STAT1 with its phosphatase, Src-homology 2-domain containing protein tyrosine phosphatase-1 (SHP-1), thereby blocking the STAT1 pathway [131] (Fig. 2D). The lack of DJ-1 results in excessive and prolonged phosphorylation of STAT1, ultimately inducing pro-inflammatory cytokines in BV-2, primary murine microglia and astrocytes [131]. Additionally, the release of IFNγ and interferon-inducible T-cell alpha chemoattractant are elevated in the SN of the DJ-1 KO mouse and DJ-1 KO microglia at baseline compared to control mice [132]. Following one-time 1 µg intra-nigral LPS administration, these cytokines were tremendously increased at day five in DJ-1 KO compared to WT mice [132], thus suggesting that DJ-1 deficiency favors the production of inflammatory mediators, both at baseline and after intra-nigral LPS injection. Of note, a clear reduction of TH positive neurons by down to 43% in DJ-1 KO mice compared to 19% in WT 5 days after a local injection of LPS into the SN was reported [132]. These results show that inflammation-induced loss of DA neurons is amplified in the DJ-1 KO mouse model (Box 3). However, these outcomes are difficult to translate to a human model as the method of intranigral injection of LPS is highly invasive and it is not directly relatable to PD onset and progression.

Interestingly, it has been recently demonstrated that DJ-1 represses the nuclear factor kappa light chain enhancer of B cells (NFκB) pathway. NFκB is a transcription factor mediating various adaptive and innate immune functions as well as acting as a pivotal mediator of inflammation (Box 2). Recently, it has been demonstrated that in the cytoplasm, DJ-1 can bind p65, a component of NFκB, in vitro in murine BV-2 microglia. By sequestering p65 in the cytoplasm, DJ-1 inhibits its propensity to over activate the NFκB pathway (Fig. 2E). When the PD-related mutation L166P was introduced into BV-2 cells, it led to the translocation of DJ-1 from the cytoplasm to the mitochondria [133]. As DJ-1 was no longer found in the cytoplasm it promoted the nuclear translocation of p65 as it facilitated the dissociation between p65 and IκBα (the inhibitor of NFκB), ultimately leading to NFκB activation [133]. Thus, NFκB was induced and activated following the lack of binding of DJ-1 to p65 and the dissociation between p65 and IκBα in the cytoplasm, which promote nuclear translocation and NFκB activation [133]. This explains the various studies showing that knockdown of DJ-1 potentiates pro-inflammatory responses in cultured murine microglia and astrocytes following activation via dopamine, LPS or IFN-γ [111, 114, 131].

Notwithstanding, the large majority of the studies that elucidated the molecular and cellular consequences of DJ-1 deficiency in microglia are based on murine culture systems (Table 1), which evidently do not take into account the cues from the CNS environment. Similar studies conducted in human cell models or patients are rare, but will be an essential future direction for translational therapeutic progresses. Taken together, in the absence of DJ-1, microglia upregulate pro-inflammatory cytokines and downregulate anti-inflammatory pathways, thereby its loss of function may increase the risk of developing PD by enhancing neuroinflammatory pathways in microglia [116, 131–133].

## Perspectives for patient management in Parkinson’s disease

### DJ-1 as a biomarker for PD diagnosis and prognosis

The only current treatment for PD is symptomatic and it is based on pharmacological dopamine supply of L-3,4-dihydroxyphenylalanine (L-DOPA) for PD patients. When the medicine is effectively managed, it can lead to a continuous control of symptoms and a sustained quality of life for, at least, the first symptomatic stages [134] (Box 1). However, 40% of PD patients receiving L-DOPA develop motor complications and fluctuations, such as L-DOPA induced dyskinesia (LID), after 4–6 years of treatment [135, 136]. Hence, there is an unmet need for treating these patients. Although sparse data is available, it appears that 40–70% of DA neurons are already degenerated at the time of diagnosis [137–139], arguing that a diagnosis of PD at an earlier stage is a prerequisite for the success of therapeutics [140]. It is therefore critical to find ways to diagnose PD in its prodromal phases (Box 1) to be able to prevent further demise of DA neurons. In this context, it is important to find early stage susceptibility risk biomarkers for PD, diagnostic and prognostic biomarkers to be able to diagnose at earlier disease stages [141].

Neuropathological studies of PD patients have shown inconsistent results on the usage of DJ-1 as a biomarker for this disease. While increased expression levels of DJ-1 in the brain (surrounding Lewy bodies), in CSF and in plasma of PD patients compared to healthy controls have been described [142–145], other studies show no difference or even decreased levels of DJ-1 in the brain of PD patients [146, 147]. Various studies of post-mortem brain tissue and plasma demonstrate that DJ-1 is oxidized in various PD patients compared to controls (Fig. 1C) [65, 142, 145, 148–150]. This observation was validated in vitro using induced pluripotent stem cell (iPSC)-derived DA neurons from DJ-1 deficient patients as well as a partial inactivation of DJ-1 in iPD neurons [149]. In addition, a positive correlation between the concentration of DJ-1 from plasma-derived neural exosomes and PD-symptoms in PD patients has been observed, although this study was based on a small cohort [151]. However, results of the measurement of DJ-1 in plasma of patient cohorts were found to demonstrate inconsistent results. This can be explained by the fact that erythrocytes are a major source of DJ-1 protein and a slight hemolysis can considerably affect measurements and obscures results, making DJ-1 from plasma an unreliable diagnostic and prognostic biomarker [143, 152, 153]. Interestingly, two studies of PD patients showed that DJ-1 in saliva is associated with disease progression, however further validation will be necessary before its usage as a prognostic biomarker [154, 155]. Furthermore, PD patients show a positive correlation between small molecule RNAs regulating DJ-1 expression found in the saliva and disease progression [156]. Further, the concentration of DJ-1 in urinary exosomes increased with age in PD patients and oxidized DJ-1 levels were two times higher in PD patients compared to healthy controls (Fig. 1C), making oxidized DJ-1 a promising biomarker in urine [157, 158]. Collectively, these studies show that oxidized DJ-1 may be a potential biomarker for PD, although further work needs to be done to clarify the most reliable source to be used in the clinic in the future [30, 159].

### Therapeutical inhibition of NLRP3 inflammasome and cGAS/STING pathways via DJ-1 in Parkinson’s disease

The NLRP3 inflammasome pathway is one of the dysregulated pathways in DJ-1-deficient microglia [127]. Of note, inflammasome activation is not only seen in patients and models of this rare recessive autosomal mutation in *PARK7.* Convincing evidence now links NLRP3 activation to PD and neurodegeneration supporting microglia and BMDM activation both in various mouse models and human patient samples [123, 124, 127, 129]. Patients and mouse models with mutations in *PINK1* and parkin (*PARK2*) also showed an over activation of the NLRP3 inflammasome [160]. Furthermore, a recent study using induced pluripotent stem cell-derived microglia from iPD patients showed an upregulation of IL-1β and NLRP3, both at the mRNA and protein levels, when compared to healthy controls [161], which confirm a study finding a high expression of NLRP3 in activated microglia as well as elevated apoptosis-associated speck-like protein containing a caspase recruitment domain 1 (ASC1) and caspase-1 in the SN of post-mortem PD brains [122]. Additionally, there is a positive correlation between the levels of NLRP3 and IL-1β in the plasma, brain and peripheral blood mononuclear cells and disease severity in iPD patients [122, 162]. The orally delivered brain-penetrant NLRP3 inhibitor MCC950 protects against neurodegeneration in various pre-clinical PD models. Recently, a phase I trial, investigating two NLRP3 inhibitors, NT-0796 and NT-0249, was successfully completed and this could be an opportunity to see similar therapeutic effects of these inhibitors in PD patients. On a similar note, the blockade of the downstream cytokine signaling of NLRP3 by administering IL-1Ra, blocking the IL-1 receptor from binding IL-1β, resulted in decreased numbers of activated microglia in the SN, less DA neuronal loss and improved motor symptoms in the MPTP mouse model of PD [129].

Dopamine can block α-syn-activated NLRP3 and IL-1β release [163]. When microglia take up α-syn it leads to potassium efflux, which activates the NLRP3 complex. This induces a toxicity-induced release of dopamine from surrounding DA neurons. This process creates a balance in the normal brain of activation with subsequent de-activation of microglia. However, the dopamine signal that dampens the NLRP3 activation in microglia is lost in PD due to the loss of DA neurons, which ultimately will activate the microglial inflammasome, resulting in neuroinflammation and neuronal loss [126, 163]. This mechanism could also explain previous findings, where dopamine exerted a dampening effect on microglia activation and cytokine secretion [164]. Additional innate immune signaling pathways, including the cyclic GMP AMP synthase (cGAS) stimulator of interferon genes (STING) cGAS/STING pathway, are activated in *PINK1* and parkin (*PARK2*) mutation models [165]. Interestingly, STING negatively correlates with DJ-1, as RNA-sequencing data of 72 patients showed high levels of STING and low levels of DJ-1 and NRF2 in the SN [166]. Withaferin A, an inhibitor of the cGAS/STING pathway, protected against gliosis and neuronal loss in the MPTP-model of PD in a DJ-1 dependent manner (Table 2) [166]. However, the elucidation of the mechanisms underlying the dampening of STING by DJ-1 would require additional studies.

**Table 2:** Immunotherapies targeting DJ-1-related neuroinflammatory pathways in Parkinson’s disease

Compound	Target	Phase	Mechanism of action and model	References
Therapeutical inhibition of NLRP3 inflammasome, cGAS/STING and NFkB in Parkinson’s disease				
MCC950	NLRP3 inflammasome inhibitor	Pre-clinical	Oral administration of MCC950 to 6-OHDA mouse model of PD and pre-formed fibril (PFF) mouse model of PD showed improved motor functions and prevented loss of dopamine and its metabolites	[122]
Nilotinib (Tasigna®, AMN107)	Inhibits microglia-mediated neuroinflammation via NFκB Tyrosine kinase inhibitor	Proof of concept in vitro and in vivo	LPS induced neurodegeneration in BV-2 cells and mouse	[199]
	Inhibits microglia-mediated neuroinflammation via NFκB Tyrosine kinase inhibitor	Clinical trial Phase II double+-blind placebo-controlled study	Long term safety in PD patients after subcutaneous injection of 150 mg/kg and 300 mg/kg nilotinib	[200]
NPT520-34	Reduces α-syn accumulation and upregulation of microtubule-associated protein 1A/1B light chain 3, a central protein in the autophagy pathway (referred to as LC3)	Pre-clinical Phase I clinical trial NCT03954600	Anti-inflammatory effects in wildtype LPS-challenged mice. Decrease α-syn load in brain and increase LC3 protein abundance and improve motor symptoms in mouse line 61	[201] NCT03954600
Dimethyl fumarate	NRF2 enhancer Decreased microgliosis and astrogliosis	Proof of concept in mouse model	NRF2 KO mice Pretreatment with dimethyl fumarate prevented neurodegeneration in 6-OHDA mouse model	[202] [203]
DJ-1 enhancers and stabilizers				
UCP0045037/compound A	DJ-1 enhancer. Binding C106	Pre-clinical Proof of concept	DJ-1 protected against neuronal degeneration in the rotenone model 6-OHDA model Better behavioral outcomes, less neuronal loss in SN	[169] [171]
UCP0054278/compound B			UCP0054278 treatment protected against neuronal death in a concentration-dependent manner in both 6-OHDA and rotenone model	[170]
Compound-23	Binding of DJ-1 and promoting neuroprotective effects of DJ-1 by preventing excessive oxidation of DJ-1 and keeping it in its active form Found via zinc compound library screening	Pre-clinical Proof of concept	Protective against PD (Rotenone and 6-OHDA) and middle cerebral artery occlusion (MCAO), stroke model Had better neuroprotective effects than compound B MPTP model. Rescued rotarod retention and neuronal loss in SN	[204] [205]
ND-13 A DJ-1 peptide	ND-13 A DJ-1 peptide	Pre-clinical Proof of concept	By preventing the oxidation of DJ-1 and maintaining the reduced DJ-1 it inhibits oxidative stress-induced toxicity Can cross the BBB and also works after subcutaneous injection	[206]
Phenyl butyrate	Neuroprotective Upregulates DJ-1	Pilot study in animals	Improving outcomes in the MPTP mouse model of PD	[175]
Withaferin A	Upregulates DJ-1 and NRF2 via STING Data extracted from patient data	Proof of concept in a small group of animals	Withaferin A treatment in MPTP-treated mice upregulated DJ-1 and NRF2 and suppressed STING, thereby protecting against DA neuronal loss	[166]

The NLRP3 and the cGAS/STING pathways are both triggered by mitochondrial dysfunction [167]. Mitochondrial DNA (mtDNA) can trigger these pathways and, not surprisingly, abnormalities in circulating cell free mtDNA have been found in PD patient cohorts [168]. The dysregulation of both innate immune pathways, NLRP3 and cGAS/STING, occurs in both idiopathic and genetic PD patients, including various models of DJ-1 deficiency. Both pathways could be promising new targets in PD and clinical trials are ongoing (Table 2). Thus, although there is still a long way to find a cure for PD, it is at least encouraging that these dysregulated neuroinflammatory pathways could potentially be targeted in various pre-clinical studies and in a broad range of PD patients (Table 2). Whether enhancing DJ-1 could dampen these dysregulated neuroinflammatory pathways would be an interesting future direction and could be a potential therapeutic.

### DJ-1 promoting compounds as promising therapeutics in Parkinson’s disease

DJ-1 promoting compounds aim to either directly upregulate DJ-1 protein or bind to non-functional over-oxidized DJ-1 protein re-establishing its function (Table 2). These therapeutics are used mainly for their capacity to limit cell death as a consequence of high oxidative stress. Pre-clinical studies showed that administration of recombinant DJ-1 protein can protect against nigral degeneration in a rat model of PD [169]. In this work, WT but not L166P DJ-1 can improve PD phenotype via protecting DA neurons against oxidative stress [169]. Various studies also showed DJ-1’s therapeutic effect in ischemic stroke models, where four different DJ-1 targeting compounds reduced the infarct volume [170–172], as well as showing recovery of cognitive functions in AD models [173]. Various compounds binding to DJ-1, including UCP0045037/compound A, UCP0054278/compound B, compound-23 and ND-3 (a DJ-1 binding peptide) were found via in silico virtual screening based on DJ-1 protein 3D structure. These compounds protected against DA neuron degeneration and restored behavioral effects in PD models by interacting with endogenous DJ-1 [174] (Table 2). Lastly, phenylbutyrate (PB) upregulates DJ-1, which in turns support the production of GSH via glutamate cysteine ligase [97, 175]. The upregulation of DJ-1 induces also the expression levels of the heat shock protein 70, which is an important protein inhibiting α-syn oligomer formation [175]. An interesting rescue experiment investigating the L199P mutation causing DJ-1 deficiency showed significantly higher tyrosine hydroxylase levels in neurons derived from iPSCs after treating with PB [176]. Although PB would not be relevant for treating patients with mutations leading to a non-functional DJ-1 protein, it could be an important therapeutic in iPD patients, both because of the neuroprotective roles of DJ-1 and as it might replace its oxidized form [65, 142]. However, these studies have only addressed the effect of these compounds on neurons, thus it is not known whether DJ-1 binding compounds might also modulate the astrocytic or microglial activation and their interplay with neurons. Additionally, it is not yet known whether DJ-1 enhancers affect the NLRP3 and cGAS/STING pathways (Table 2). Notwithstanding, detailed mechanistic insights into how DJ-1 enhancers exert their therapeutic effects are still missing. Most of these compounds targeting DJ-1 and enhancing its effect can cross the BBB and have potent neuroprotective effects (Table 2). It will be interesting to see if the outcome of future clinical studies using these compounds will recapitulate the promising results obtained in rodent models. Further, these studies have only addressed the effect of these compounds on neurons, thus it is not known whether DJ-1 promoting compounds might also modulate the astrocytic or microglial activation and their interplay with neurons. Additionally, it is not yet known whether DJ-1 enhancers affect the NLRP3 and cGAS/STING pathways (Table 2).

## Conclusions and perspectives

The activation of microglia was discovered decades ago in post-mortem PD brains and has since then been confirmed in various studies in cell and animal models of PD as well as in PD patients. Mutations in *PARK7* leading to non-functional or complete loss of DJ-1 protein lead to early-onset PD with autosomal recessive inheritance. Overall, PARK7/DJ-1 dysfunctions have a prominent effect on microglia immune responses and neuroinflammatory processes. Several similarities are found between iPD and genetic PD patient groups, including the neuroprotective functions of DJ-1. In this context, as DJ-1 is oxidized in various PD patients, iPD cases might show an immune phenocopy of DJ-1 deficient patients. Therapeutics elevating DJ-1 levels in PD animal models show promising results and would need to be translated in the clinic. Future studies aimed at investigating more specifically the link between CNS inflammatory pathways, including oxidative pathways and cGAS/STING-, NFκB- and NLRP3-derived signaling, and neurodegenerative processes may reveal molecular mechanisms to translate the mechanistic insight of dysregulated immune pathways to therapeutic targets in a larger group of PD patients. The study of mutations leading to PD contributes to our understanding of the cellular pathways underlying the neurodegenerative outcomes and may pave the way to the development of novel therapeutics promoting neuroprotection and dampening microglia activation in PD patients.

## Data Availability

Not applicable.

## Abbreviations

AD: Alzheimer’s disease
ASC: Apoptosis-associated speck-like protein containing a caspase recruitment domain
A-syn: Alpha-synuclein
ARE: Antioxidant response element
BBB: Blood–brain barrier
BDNF: Brain-derived neurotrophic factor
BMDM: Bone marrow-derived macrophage
cGAS: Cyclic GMP AMP synthase
CHAC1: Glutathione-specific gamma-glutamyl cyclotransferase, CNS: Central nervous system
CSF: Cerebrospinal fluid
DA: Dopaminergic
DAM: Disease-associated microglia
DJ-1: Deglycase 1
EOPD: Early-onset Parkinson’s disease
GSH: Glutathione
IFN: Interferon
iPD: Idiopathic Parkinson’s disease
iPSC: Induced pluripotent stem cell
JAK–STAT: Janus kinase/signal transducer and activator of transcription
KEAP-1: Kelch-like ECH protein 1
L-DOPA: L-3,4-Dihydroxyphenylalanine
LID: L-DOPA-induced dyskinesia
LOPD: Late-onset Parkinson’s disease
LPS: Lipopolysaccharide
LRRK2: Leucine rich repeat kinase 2
MPTP: 1-Methyl-4-phenyl-1,2,3,6-tetrahydropyridine
NADPH: Nicotinamide adenine dinucleotide phosphate
NFκB: Nuclear factor kappa light chain enhancer of B cells
NLR: Nucleotide-binding leucine-rich repeat receptor
NLRP3: NLR pyrin domain containing 3/NOD like receptor protein
NRF2: NF-E2 related factor 2
NO: Nitric oxide
PAMP: Pathogen-associated molecular pattern
PB: Phenyl butyrate
PD: Parkinson’s disease
PRR: Pathogen recognition receptor
ROS: Reactive oxygen species
SHP-1: Src-homology 2-domain containing protein tyrosine phosphatase-1
SN: Substantia nigra
STING: Stimulator of interferon genes
TH: Tyrosine hydroxylase
TLR-4: Toll-like receptor 4
TNF: Tumor necrosis factor
TRX1: Thioredoxin-1
WT: Wild type
YOPD: Young-onset Parkinson’s disease

## References

[1] Mittelbronn M, Dietz K, Schluesener HJ, Meyermann R (2001). Local distribution of microglia in the normal adult human central nervous system differs by up to one order of magnitude. Acta Neuropathol.

[2] Lawson LJ, Perry VH, Dri P, Gordon S (1990). Heterogeneity in the distribution and morphology of microglia in the normal adult mouse brain. Neuroscience.

[3] Sousa C, Biber K, Michelucci A (2017). Cellular and molecular characterization of microglia: a unique immune cell population. Front Immunol.

[4] Hickman SE, Kingery ND, Ohsumi TK, Borowsky ML, Wang LC, Means TK (2013). The microglial sensome revealed by direct RNA sequencing. Nat Neurosci.

[5] Kettenmann H, Hanisch UK, Noda M, Verkhratsky A (2011). Physiology of microglia. Physiol Rev.

[6] Parkhurst CN, Yang G, Ninan I, Savas JN, Yates JR, Lafaille JJ (2013). Microglia promote learning-dependent synapse formation through brain-derived neurotrophic factor. Cell.

[7] Kierdorf K, Prinz M (2017). Microglia in steady state. J Clin Invest.

[8] Hagemeyer N, Hanft KM, Akriditou MA, Unger N, Park ES, Stanley ER (2017). Microglia contribute to normal myelinogenesis and to oligodendrocyte progenitor maintenance during adulthood. Acta Neuropathol.

[9] Miron VE (2017). Microglia-driven regulation of oligodendrocyte lineage cells, myelination, and remyelination. J Leukoc Biol.

[10] Paolicelli RC, Sierra A, Stevens B, Tremblay ME, Aguzzi A, Ajami B (2022). Microglia states and nomenclature: a field at its crossroads. Neuron.

[11] Chen Y, Colonna M (2021). Microglia in Alzheimer's disease at single-cell level. Are there common patterns in humans and mice?. J Exp Med.

[12] de Lau LM, Breteler MM (2006). Epidemiology of Parkinson's disease. Lancet Neurol.

[13] Dorsey ER, Bloem BR (2018). The Parkinson pandemic-a call to action. JAMA Neurol.

[14] Goetz CG (2011). The history of Parkinson's disease: early clinical descriptions and neurological therapies. Cold Spring Harb Perspect Med.

[15] Parkinson J (2002). An essay on the shaking palsy. J Neuropsychiatry Clin Neurosci.

[16] Lees AJ, Hardy J, Revesz T (2009). Parkinson's disease. Lancet.

[17] Poewe W, Seppi K, Tanner CM, Halliday GM, Brundin P, Volkmann J (2017). Parkinson disease. Nat Rev Dis Primers.

[18] Smeyne RJ, Noyce AJ, Byrne M, Savica R, Marras C (2021). Infection and risk of Parkinson's disease. J Parkinsons Dis.

[19] Schapira AH, Jenner P (2011). Etiology and pathogenesis of Parkinson's disease. Mov Disord.

[20] Farrow SL, Cooper AA, O'Sullivan JM (2022). Redefining the hypotheses driving Parkinson's diseases research. NPJ Parkinsons Dis.

[21] Islam MS, Azim F, Saju H, Zargaran A, Shirzad M, Kamal M (2021). Pesticides and Parkinson's disease: current and future perspective. J Chem Neuroanat.

[22] Dorsey ER, Constantinescu R, Thompson JP, Biglan KM, Holloway RG, Kieburtz K (2007). Projected number of people with Parkinson disease in the most populous nations, 2005 through 2030. Neurology.

[23] Deng H, Wang P, Jankovic J (2018). The genetics of Parkinson disease. Ageing Res Rev.

[24] Nalls MA, Blauwendraat C, Vallerga CL, Heilbron K, Bandres-Ciga S, Chang D (2019). Identification of novel risk loci, causal insights, and heritable risk for Parkinson's disease: a meta-analysis of genome-wide association studies. Lancet Neurol.

[25] Germer EL, Imhoff S, Vilarino-Guell C, Kasten M, Seibler P, Bruggemann N (2019). The role of rare coding variants in Parkinson's disease GWAS loci. Front Neurol.

[26] Simon-Sanchez J, Schulte C, Bras JM, Sharma M, Gibbs JR, Berg D (2009). Genome-wide association study reveals genetic risk underlying Parkinson's disease. Nat Genet.

[27] Nalls MA, Pankratz N, Lill CM, Do CB, Hernandez DG, Saad M (2014). Large-scale meta-analysis of genome-wide association data identifies six new risk loci for Parkinson's disease. Nat Genet.

[28] Schiesling C, Kieper N, Seidel K, Kruger R (2008). Review: familial Parkinson's disease–genetics, clinical phenotype and neuropathology in relation to the common sporadic form of the disease. Neuropathol Appl Neurobiol.

[29] Andreeva A, Bekkhozhin Z, Omertassova N, Baizhumanov T, Yeltay G, Akhmetali M (2019). The apparent deglycase activity of DJ-1 results from the conversion of free methylglyoxal present in fast equilibrium with hemithioacetals and hemiaminals. J Biol Chem.

[30] Huang M, Chen S (2021). DJ-1 in neurodegenerative diseases: pathogenesis and clinical application. Prog Neurobiol.

[31] Zeng N, Capelle CM, Baron A, Kobayashi T, Cire S, Tslaf V (2022). DJ-1 depletion prevents immunoaging in T-cell compartments. EMBO Rep.

[32] Danileviciute E, Zeng N, Capelle CM, Paczia N, Gillespie MA, Kurniawan H (2022). PARK7/DJ-1 promotes pyruvate dehydrogenase activity and maintains T(reg) homeostasis during ageing. Nat Metab.

[33] Tansey MG, Wallings RL, Houser MC, Herrick MK, Keating CE, Joers V (2022). Inflammation and immune dysfunction in Parkinson disease. Nat Rev Immunol.

[34] Antony PM, Diederich NJ, Kruger R, Balling R (2013). The hallmarks of Parkinson's disease. FEBS J.

[35] Badanjak K, Fixemer S, Smajic S, Skupin A, Grunewald A (2021). The Contribution of microglia to neuroinflammation in Parkinson’s disease. Int J Mol Sci.

[36] Block ML, Zecca L, Hong JS (2007). Microglia-mediated neurotoxicity: uncovering the molecular mechanisms. Nat Rev Neurosci.

[37] Le W, Wu J, Tang Y (2016). Protective microglia and their regulation in Parkinson's disease. Front Mol Neurosci.

[38] McGeer PL, Itagaki S, Boyes BE, McGeer EG (1988). Reactive microglia are positive for HLA-DR in the substantia nigra of Parkinson's and Alzheimer's disease brains. Neurology.

[39] Hirsch EC, Vyas S, Hunot S (2012). Neuroinflammation in Parkinson's disease. Parkinsonism Relat Disord.

[40] Smajic S, Prada-Medina CA, Landoulsi Z, Ghelfi J, Delcambre S, Dietrich C (2022). Single-cell sequencing of human midbrain reveals glial activation and a Parkinson-specific neuronal state. Brain.

[41] Imamura K, Hishikawa N, Sawada M, Nagatsu T, Yoshida M, Hashizume Y (2003). Distribution of major histocompatibility complex class II-positive microglia and cytokine profile of Parkinson's disease brains. Acta Neuropathol.

[42] Langston JW, Forno LS, Tetrud J, Reeves AG, Kaplan JA, Karluk D (1999). Evidence of active nerve cell degeneration in the substantia nigra of humans years after 1-methyl-4-phenyl-1,2,3,6-tetrahydropyridine exposure. Ann Neurol.

[43] Gerhard A, Pavese N, Hotton G, Turkheimer F, Es M, Hammers A (2006). In vivo imaging of microglial activation with [11C](R)-PK11195 PET in idiopathic Parkinson's disease. Neurobiol Dis.

[44] Iannaccone S, Cerami C, Alessio M, Garibotto V, Panzacchi A, Olivieri S (2013). In vivo microglia activation in very early dementia with Lewy bodies, comparison with Parkinson's disease. Parkinsonism Relat Disord.

[45] Berg D, Godau J, Riederer P, Gerlach M, Arzberger T (2010). Microglia activation is related to substantia nigra echogenicity. J Neural Transm (Vienna).

[46] Doorn KJ, Moors T, Drukarch B, van de Berg W, Lucassen PJ, van Dam AM (2014). Microglial phenotypes and toll-like receptor 2 in the substantia nigra and hippocampus of incidental Lewy body disease cases and Parkinson's disease patients. Acta Neuropathol Commun.

[47] Miklossy J, Doudet DD, Schwab C, Yu S, McGeer EG, McGeer PL (2006). Role of ICAM-1 in persisting inflammation in Parkinson disease and MPTP monkeys. Exp Neurol.

[48] Mogi M, Harada M, Kondo T, Riederer P, Inagaki H, Minami M (1994). Interleukin-1 beta, interleukin-6, epidermal growth factor and transforming growth factor-alpha are elevated in the brain from parkinsonian patients. Neurosci Lett.

[49] Mogi M, Harada M, Kondo T, Riederer P, Nagatsu T (1996). Interleukin-2 but not basic fibroblast growth factor is elevated in parkinsonian brain. J Neural Transm (Vienna).

[50] Wijeyekoon RS, Moore SF, Farrell K, Breen DP, Barker RA, Williams-Gray CH (2020). Cerebrospinal fluid cytokines and neurodegeneration-associated proteins in Parkinson's disease. Mov Disord.

[51] Chen X, Hu Y, Cao Z, Liu Q, Cheng Y (2018). Cerebrospinal fluid inflammatory cytokine aberrations in Alzheimer's disease, Parkinson's disease and amyotrophic lateral sclerosis: a systematic review and meta-analysis. Front Immunol.

[52] Vawter MP, Dillon-Carter O, Tourtellotte WW, Carvey P, Freed WJ (1996). TGFbeta1 and TGFbeta2 concentrations are elevated in Parkinson's disease in ventricular cerebrospinal fluid. Exp Neurol.

[53] Bido S, Muggeo S, Massimino L, Marzi MJ, Giannelli SG, Melacini E (2021). Microglia-specific overexpression of alpha-synuclein leads to severe dopaminergic neurodegeneration by phagocytic exhaustion and oxidative toxicity. Nat Commun.

[54] Scheiblich H, Dansokho C, Mercan D, Schmidt SV, Bousset L, Wischhof L (2021). Microglia jointly degrade fibrillar alpha-synuclein cargo by distribution through tunneling nanotubes. Cell.

[55] Glass CK, Saijo K, Winner B, Marchetto MC, Gage FH (2010). Mechanisms underlying inflammation in neurodegeneration. Cell.

[56] Lotharius J, Brundin P (2002). Pathogenesis of Parkinson's disease: dopamine, vesicles and alpha-synuclein. Nat Rev Neurosci.

[57] Jenner P (2003). Oxidative stress in Parkinson's disease. Ann Neurol.

[58] Wang X, Michaelis EK (2010). Selective neuronal vulnerability to oxidative stress in the brain. Front Aging Neurosci.

[59] Dorszewska J, Kowalska M, Prendecki M, Piekut T, Kozlowska J, Kozubski W (2021). Oxidative stress factors in Parkinson's disease. Neural Regen Res.

[60] Kim WG, Mohney RP, Wilson B, Jeohn GH, Liu B, Hong JS (2000). Regional difference in susceptibility to lipopolysaccharide-induced neurotoxicity in the rat brain: role of microglia. J Neurosci.

[61] Uriarte Huarte O, Kyriakis D, Heurtaux T, Pires-Afonso Y, Grzyb K, Halder R (2021). Single-cell transcriptomics and in situ morphological analyses reveal microglia heterogeneity across the nigrostriatal pathway. Front Immunol.

[62] Gao HM, Jiang J, Wilson B, Zhang W, Hong JS, Liu B (2002). Microglial activation-mediated delayed and progressive degeneration of rat nigral dopaminergic neurons: relevance to Parkinson's disease. J Neurochem.

[63] Bader V, Ran Zhu X, Lubbert H, Stichel CC (2005). Expression of DJ-1 in the adult mouse CNS. Brain Res.

[64] Zhang L, Shimoji M, Thomas B, Moore DJ, Yu SW, Marupudi NI (2005). Mitochondrial localization of the Parkinson's disease related protein DJ-1: implications for pathogenesis. Hum Mol Genet.

[65] Bandopadhyay R, Kingsbury AE, Cookson MR, Reid AR, Evans IM, Hope AD (2004). The expression of DJ-1 (PARK7) in normal human CNS and idiopathic Parkinson's disease. Brain.

[66] Nagakubo D, Taira T, Kitaura H, Ikeda M, Tamai K, Iguchi-Ariga SM (1997). DJ-1, a novel oncogene which transforms mouse NIH3T3 cells in cooperation with ras. Biochem Biophys Res Commun.

[67] van Duijn CM, Dekker MC, Bonifati V, Galjaard RJ, Houwing-Duistermaat JJ, Snijders PJ (2001). Park7, a novel locus for autosomal recessive early-onset parkinsonism, on chromosome 1p36. Am J Hum Genet.

[68] Bonifati V, Rizzu P, van Baren MJ, Schaap O, Breedveld GJ, Krieger E (2003). Mutations in the DJ-1 gene associated with autosomal recessive early-onset parkinsonism. Science.

[69] Gibson R, Dalvi SP, Dalvi PS (2021). DJ-1 and Parkinson's disease. Brain Disorders.

[70] Shendelman S, Jonason A, Martinat C, Leete T, Abeliovich A (2004). DJ-1 is a redox-dependent molecular chaperone that inhibits alpha-synuclein aggregate formation. PLoS Biol.

[71] Nash Y, Schmukler E, Trudler D, Pinkas-Kramarski R, Frenkel D (2017). DJ-1 deficiency impairs autophagy and reduces alpha-synuclein phagocytosis by microglia. J Neurochem.

[72] Junn E, Taniguchi H, Jeong BS, Zhao X, Ichijo H, Mouradian MM (2005). Interaction of DJ-1 with Daxx inhibits apoptosis signal-regulating kinase 1 activity and cell death. Proc Natl Acad Sci USA.

[73] Narayan S, Liew Z, Bronstein JM, Ritz B (2017). Occupational pesticide use and Parkinson's disease in the Parkinson environment gene (PEG) study. Environ Int.

[74] Mathas N, Poncet G, Laurent C, Larigot L, Le-Grand B, Gonis E (2023). Inhibition by pesticides of the DJ-1/Park7 protein related to Parkinson disease. Toxicology.

[75] Goldman SM (2014). Environmental toxins and Parkinson's disease. Annu Rev Pharmacol Toxicol.

[76] Tanner CM, Kamel F, Ross GW, Hoppin JA, Goldman SM, Korell M (2011). Rotenone, paraquat, and Parkinson's disease. Environ Health Perspect.

[77] Bjorkblom B, Adilbayeva A, Maple-Grodem J, Piston D, Okvist M, Xu XM (2013). Parkinson disease protein DJ-1 binds metals and protects against metal-induced cytotoxicity. J Biol Chem.

[78] Pyatha S, Kim H, Lee D, Kim K (2022). Association between heavy metal exposure and Parkinson's disease: a review of the mechanisms related to oxidative stress. Antioxidants (Basel)..

[79] Wang A, Costello S, Cockburn M, Zhang X, Bronstein J, Ritz B (2011). Parkinson's disease risk from ambient exposure to pesticides. Eur J Epidemiol.

[80] De Miranda BR, Goldman SM, Miller GW, Greenamyre JT, Dorsey ER (2022). Preventing Parkinson's disease: an environmental agenda. J Parkinsons Dis.

[81] Dorsey ER, Zafar M, Lettenberger SE, Pawlik ME, Kinel D, Frissen M (2023). Trichloroethylene: an invisible cause of Parkinson's disease?. J Parkinsons Dis.

[82] Khan Z, Ali SA (2018). Oxidative stress-related biomarkers in Parkinson's disease: a systematic review and meta-analysis. Iran J Neurol.

[83] Mailloux RJ (2020). An update on mitochondrial reactive oxygen species production. Antioxidants (Basel)..

[84] Babior BM (2000). Phagocytes and oxidative stress. Am J Med.

[85] Haslund-Vinding J, McBean G, Jaquet V, Vilhardt F (2017). NADPH oxidases in oxidant production by microglia: activating receptors, pharmacology and association with disease. Br J Pharmacol.

[86] Canton M, Sanchez-Rodriguez R, Spera I, Venegas FC, Favia M, Viola A (2021). Reactive oxygen species in macrophages: sources and targets. Front Immunol.

[87] Keeney MT, Hoffman EK, Farmer K, Bodle CR, Fazzari M, Zharikov A (2022). NADPH oxidase 2 activity in Parkinson's disease. Neurobiol Dis.

[88] Sumi N, Nishioku T, Takata F, Matsumoto J, Watanabe T, Shuto H (2010). Lipopolysaccharide-activated microglia induce dysfunction of the blood-brain barrier in rat microvascular endothelial cells co-cultured with microglia. Cell Mol Neurobiol.

[89] Zhang W, Wang T, Pei Z, Miller DS, Wu X, Block ML (2005). Aggregated alpha-synuclein activates microglia: a process leading to disease progression in Parkinson's disease. FASEB J.

[90] Clements CM, McNally RS, Conti BJ, Mak TW, Ting JP (2006). DJ-1, a cancer- and Parkinson's disease-associated protein, stabilizes the antioxidant transcriptional master regulator Nrf2. Proc Natl Acad Sci USA.

[91] Canet-Aviles RM, Wilson MA, Miller DW, Ahmad R, McLendon C, Bandyopadhyay S (2004). The Parkinson's disease protein DJ-1 is neuroprotective due to cysteine-sulfinic acid-driven mitochondrial localization. Proc Natl Acad Sci USA.

[92] Taira T, Saito Y, Niki T, Iguchi-Ariga SM, Takahashi K, Ariga H (2004). DJ-1 has a role in antioxidative stress to prevent cell death. EMBO Rep.

[93] Wilson MA (2011). The role of cysteine oxidation in DJ-1 function and dysfunction. Antioxid Redox Signal.

[94] Junn E, Jang WH, Zhao X, Jeong BS, Mouradian MM (2009). Mitochondrial localization of DJ-1 leads to enhanced neuroprotection. J Neurosci Res.

[95] Hayashi T, Ishimori C, Takahashi-Niki K, Taira T, Kim YC, Maita H (2009). DJ-1 binds to mitochondrial complex I and maintains its activity. Biochem Biophys Res Commun.

[96] Sekito A, Koide-Yoshida S, Niki T, Taira T, Iguchi-Ariga SM, Ariga H (2006). DJ-1 interacts with HIPK1 and affects H2O2-induced cell death. Free Radic Res.

[97] Zhou W, Freed CR (2005). DJ-1 up-regulates glutathione synthesis during oxidative stress and inhibits A53T alpha-synuclein toxicity. J Biol Chem.

[98] Ge Y, Zheng X, Mao S, Zhang Q, Hu G, Wei Y (2022). DJ-1 inhibits glutathione degradation by downregulating CHAC1 expression in astrocytes. Neurosci Res.

[99] Honbou K, Suzuki NN, Horiuchi M, Niki T, Taira T, Ariga H (2003). The crystal structure of DJ-1, a protein related to male fertility and Parkinson's disease. J Biol Chem.

[100] Huai Q, Sun Y, Wang H, Chin LS, Li L, Robinson H (2003). Crystal structure of DJ-1/RS and implication on familial Parkinson's disease. FEBS Lett.

[101] Tao X, Tong L (2003). Crystal structure of human DJ-1, a protein associated with early onset Parkinson's disease. J Biol Chem.

[102] Dekker MC, Eshuis SA, Maguire RP, Veenma-van der Duijn L, Pruim J, Snijders PJ (2004). PET neuroimaging and mutations in the DJ-1 gene. J Neural Transm (Vienna).

[103] Chu JS, Liu TH, Wang KL, Han CL, Liu YP, Michitomo S (2019). The metabolic activity of caudate and prefrontal cortex negatively correlates with the severity of idiopathic Parkinson's disease. Aging Dis.

[104] Braak H, Del Tredici K (2017). Neuropathological staging of brain pathology in sporadic Parkinson's disease: separating the wheat from the Chaff. J Parkinsons Dis.

[105] Taipa R, Pereira C, Reis I, Alonso I, Bastos-Lima A, Melo-Pires M (2016). DJ-1 linked parkinsonism (PARK7) is associated with Lewy body pathology. Brain.

[106] Mencke P, Boussaad I, Romano CD, Kitami T, Linster CL, Kruger R (2021). The role of DJ-1 in Cellular metabolism and pathophysiological implications for Parkinson's disease. Cells.

[107] Ariga H, Takahashi-Niki K, Kato I, Maita H, Niki T, Iguchi-Ariga SM (2013). Neuroprotective function of DJ-1 in Parkinson's disease. Oxid Med Cell Longev.

[108] Cookson MR (2010). DJ-1, PINK1, and their effects on mitochondrial pathways. Mov Disord.

[109] Dolgacheva LP, Berezhnov AV, Fedotova EI, Zinchenko VP, Abramov AY (2019). Role of DJ-1 in the mechanism of pathogenesis of Parkinson's disease. J Bioenerg Biomembr.

[110] Kim RH, Smith PD, Aleyasin H, Hayley S, Mount MP, Pownall S (2005). Hypersensitivity of DJ-1-deficient mice to 1-methyl-4-phenyl-1,2,3,6-tetrahydropyrindine (MPTP) and oxidative stress. Proc Natl Acad Sci USA.

[111] Trudler D, Weinreb O, Mandel SA, Youdim MB, Frenkel D (2014). DJ-1 deficiency triggers microglia sensitivity to dopamine toward a pro-inflammatory phenotype that is attenuated by rasagiline. J Neurochem.

[112] Liu W, Wu H, Chen L, Wen Y, Kong X, Gao WQ (2015). Park7 interacts with p47(phox) to direct NADPH oxidase-dependent ROS production and protect against sepsis. Cell Res.

[113] Amatullah H, Shan Y, Beauchamp BL, Gali PL, Gupta S, Maron-Gutierrez T (2017). DJ-1/PARK7 impairs bacterial clearance in sepsis. Am J Respir Crit Care Med.

[114] Meiser J, Delcambre S, Wegner A, Jager C, Ghelfi J, d'Herouel AF (2016). Loss of DJ-1 impairs antioxidant response by altered glutamine and serine metabolism. Neurobiol Dis.

[115] Chen W, Liu H, Liu S, Kang Y, Nie Z, Lei H (2022). Altered prefrontal neurochemistry in the DJ-1 knockout mouse model of Parkinson's disease: complementary semi-quantitative analyses with in vivo magnetic resonance spectroscopy and MALDI-MSI. Anal Bioanal Chem.

[116] Ji YJ, Wang HL, Yin BL, Ren XY (2020). Down-regulation of DJ-1 augments neuroinflammation via Nrf2/Trx1/NLRP3 Axis in MPTP-induced Parkinson's disease mouse model. Neuroscience.

[117] Hou Y, Wang Y, He Q, Li L, Xie H, Zhao Y (2018). Nrf2 inhibits NLRP3 inflammasome activation through regulating Trx1/TXNIP complex in cerebral ischemia reperfusion injury. Behav Brain Res.

[118] Im JY, Lee KW, Woo JM, Junn E, Mouradian MM (2012). DJ-1 induces thioredoxin 1 expression through the Nrf2 pathway. Hum Mol Genet.

[119] Wang S, Yuan YH, Chen NH, Wang HB (2019). The mechanisms of NLRP3 inflammasome/pyroptosis activation and their role in Parkinson's disease. Int Immunopharmacol.

[120] Baroja-Mazo A, Martin-Sanchez F, Gomez AI, Martinez CM, Amores-Iniesta J, Compan V (2014). The NLRP3 inflammasome is released as a particulate danger signal that amplifies the inflammatory response. Nat Immunol.

[121] Alboni S, Cervia D, Sugama S, Conti B (2010). Interleukin 18 in the CNS. J Neuroinflammation.

[122] Gordon R, Albornoz EA, Christie DC, Langley MR, Kumar V, Mantovani S (2018). Inflammasome inhibition prevents alpha-synuclein pathology and dopaminergic neurodegeneration in mice. Sci Transl Med..

[123] Zhou Y, Lu M, Du RH, Qiao C, Jiang CY, Zhang KZ (2016). MicroRNA-7 targets Nod-like receptor protein 3 inflammasome to modulate neuroinflammation in the pathogenesis of Parkinson's disease. Mol Neurodegener.

[124] Panicker N, Sarkar S, Harischandra DS, Neal M, Kam TI, Jin H (2019). Fyn kinase regulates misfolded alpha-synuclein uptake and NLRP3 inflammasome activation in microglia. J Exp Med.

[125] Freeman L, Guo H, David CN, Brickey WJ, Jha S, Ting JP (2017). NLR members NLRC4 and NLRP3 mediate sterile inflammasome activation in microglia and astrocytes. J Exp Med.

[126] Pike AF, Szabo I, Veerhuis R, Bubacco L (2022). The potential convergence of NLRP3 inflammasome, potassium, and dopamine mechanisms in Parkinson's disease. NPJ Parkinsons Dis.

[127] Nguyen LTN, Nguyen HD, Kim YJ, Nguyen TT, Lai TT, Lee YK (2022). Role of NLRP3 inflammasome in Parkinson's disease and therapeutic considerations. J Parkinsons Dis.

[128] Mao Z, Liu C, Ji S, Yang Q, Ye H, Han H (2017). The NLRP3 inflammasome is involved in the pathogenesis of Parkinson's disease in rats. Neurochem Res.

[129] Lee E, Hwang I, Park S, Hong S, Hwang B, Cho Y (2019). MPTP-driven NLRP3 inflammasome activation in microglia plays a central role in dopaminergic neurodegeneration. Cell Death Differ.

[130] Jain M, Singh MK, Shyam H, Mishra A, Kumar S, Kumar A (2021). Role of JAK/STAT in the neuroinflammation and its association with neurological disorders. Ann Neurosci.

[131] Kim JH, Choi DJ, Jeong HK, Kim J, Kim DW, Choi SY (2013). DJ-1 facilitates the interaction between STAT1 and its phosphatase, SHP-1, in brain microglia and astrocytes: a novel anti-inflammatory function of DJ-1. Neurobiol Dis.

[132] Chien CH, Lee MJ, Liou HC, Liou HH, Fu WM (2016). Microglia-derived cytokines/chemokines are involved in the enhancement of LPS-induced loss of nigrostriatal dopaminergic neurons in DJ-1 knockout mice. PLoS ONE.

[133] Lin Z, Chen C, Yang D, Ding J, Wang G, Ren H (2021). DJ-1 inhibits microglial activation and protects dopaminergic neurons in vitro and in vivo through interacting with microglial p65. Cell Death Dis.

[134] Teymourian H, Tehrani F, Longardner K, Mahato K, Podhajny T, Moon JM (2022). Closing the loop for patients with Parkinson disease: where are we?. Nat Rev Neurol.

[135] Ahlskog JE, Muenter MD (2001). Frequency of levodopa-related dyskinesias and motor fluctuations as estimated from the cumulative literature. Mov Disord.

[136] Jankovic J, Aguilar LG (2008). Current approaches to the treatment of Parkinson's disease. Neuropsychiatr Dis Treat.

[137] Rajput AH, Sitte HH, Rajput A, Fenton ME, Pifl C, Hornykiewicz O (2008). Globus pallidus dopamine and Parkinson motor subtypes: clinical and brain biochemical correlation. Neurology.

[138] Kordower JH, Olanow CW, Dodiya HB, Chu Y, Beach TG, Adler CH (2013). Disease duration and the integrity of the nigrostriatal system in Parkinson's disease. Brain.

[139] Damier P, Hirsch EC, Agid Y, Graybiel AM (1999). The substantia nigra of the human brain. II. Patterns of loss of dopamine-containing neurons in Parkinson's disease. Brain.

[140] Mahlknecht P, Marini K, Werkmann M, Poewe W, Seppi K (2022). Prodromal Parkinson's disease: hype or hope for disease-modification trials?. Transl Neurodegener.

[141] Tolosa E, Garrido A, Scholz SW, Poewe W (2021). Challenges in the diagnosis of Parkinson's disease. Lancet Neurol.

[142] Choi J, Sullards MC, Olzmann JA, Rees HD, Weintraub ST, Bostwick DE (2006). Oxidative damage of DJ-1 is linked to sporadic Parkinson and Alzheimer diseases. J Biol Chem.

[143] Waragai M, Nakai M, Wei J, Fujita M, Mizuno H, Ho G (2007). Plasma levels of DJ-1 as a possible marker for progression of sporadic Parkinson's disease. Neurosci Lett.

[144] Jin J, Meredith GE, Chen L, Zhou Y, Xu J, Shie FS (2005). Quantitative proteomic analysis of mitochondrial proteins: relevance to Lewy body formation and Parkinson's disease. Brain Res Mol Brain Res.

[145] Nural H, He P, Beach T, Sue L, Xia W, Shen Y (2009). Dissembled DJ-1 high molecular weight complex in cortex mitochondria from Parkinson's disease patients. Mol Neurodegener.

[146] Piston D, Alvarez-Erviti L, Bansal V, Gargano D, Yao Z, Szabadkai G (2017). DJ-1 is a redox sensitive adapter protein for high molecular weight complexes involved in regulation of catecholamine homeostasis. Hum Mol Genet.

[147] Kumaran R, Vandrovcova J, Luk C, Sharma S, Renton A, Wood NW (2009). Differential DJ-1 gene expression in Parkinson's disease. Neurobiol Dis.

[148] Blackinton J, Kumaran R, van der Brug MP, Ahmad R, Olson L, Galter D (2009). Post-transcriptional regulation of mRNA associated with DJ-1 in sporadic Parkinson disease. Neurosci Lett.

[149] Burbulla LF, Song P, Mazzulli JR, Zampese E, Wong YC, Jeon S (2017). Dopamine oxidation mediates mitochondrial and lysosomal dysfunction in Parkinson's disease. Science.

[150] Mita Y, Kataoka Y, Saito Y, Kashi T, Hayashi K, Iwasaki A (2018). Distribution of oxidized DJ-1 in Parkinson's disease-related sites in the brain and in the peripheral tissues: effects of aging and a neurotoxin. Sci Rep.

[151] Zhao ZH, Chen ZT, Zhou RL, Zhang X, Ye QY, Wang YZ (2018). Increased DJ-1 and alpha-synuclein in plasma neural-derived exosomes as potential markers for Parkinson's disease. Front Aging Neurosci.

[152] Lin X, Cook TJ, Zabetian CP, Leverenz JB, Peskind ER, Hu SC (2012). DJ-1 isoforms in whole blood as potential biomarkers of Parkinson disease. Sci Rep.

[153] Shi M, Zabetian CP, Hancock AM, Ginghina C, Hong Z, Yearout D (2010). Significance and confounders of peripheral DJ-1 and alpha-synuclein in Parkinson's disease. Neurosci Lett.

[154] Kang WY, Yang Q, Jiang XF, Chen W, Zhang LY, Wang XY (2014). Salivary DJ-1 could be an indicator of Parkinson's disease progression. Front Aging Neurosci.

[155] Devic I, Hwang H, Edgar JS, Izutsu K, Presland R, Pan C (2011). Salivary alpha-synuclein and DJ-1: potential biomarkers for Parkinson's disease. Brain.

[156] Chen Y, Zheng J, Su L, Chen F, Zhu R, Chen X (2020). Increased salivary microRNAs That regulate DJ-1 gene expression as potential markers for Parkinson's disease. Front Aging Neurosci.

[157] Ho DH, Yi S, Seo H, Son I, Seol W (2014). Increased DJ-1 in urine exosome of Korean males with Parkinson's disease. Biomed Res Int.

[158] Jang J, Jeong S, Lee SI, Seol W, Seo H, Son I (2018). Oxidized DJ-1 levels in urine samples as a putative biomarker for Parkinson's disease. Parkinsons Dis.

[159] Dos Santos MCT, Scheller D, Schulte C, Mesa IR, Colman P, Bujac SR (2018). Evaluation of cerebrospinal fluid proteins as potential biomarkers for early stage Parkinson's disease diagnosis. PLoS ONE.

[160] Mouton-Liger F, Rosazza T, Sepulveda-Diaz J, Ieang A, Hassoun SM, Claire E (2018). Parkin deficiency modulates NLRP3 inflammasome activation by attenuating an A20-dependent negative feedback loop. Glia.

[161] Badanjak K, Mulica P, Smajic S, Delcambre S, Tranchevent LC, Diederich N (2021). iPSC-derived microglia as a model to study inflammation in idiopathic Parkinson's disease. Front Cell Dev Biol.

[162] Fan Z, Pan YT, Zhang ZY, Yang H, Yu SY, Zheng Y (2020). Systemic activation of NLRP3 inflammasome and plasma alpha-synuclein levels are correlated with motor severity and progression in Parkinson's disease. J Neuroinflammation.

[163] Pike AF, Longhena F, Faustini G, van Eik JM, Gombert I, Herrebout MAC (2022). Dopamine signaling modulates microglial NLRP3 inflammasome activation: implications for Parkinson's disease. J Neuroinflammation.

[164] Carta AR, Mulas G, Bortolanza M, Duarte T, Pillai E, Fisone G (2017). l-DOPA-induced dyskinesia and neuroinflammation: do microglia and astrocytes play a role?. Eur J Neurosci.

[165] Sliter DA, Martinez J, Hao L, Chen X, Sun N, Fischer TD (2018). Parkin and PINK1 mitigate STING-induced inflammation. Nature.

[166] Zhao M, Wang B, Zhang C, Su Z, Guo B, Zhao Y (2021). The DJ1-Nrf2-STING axis mediates the neuroprotective effects of Withaferin A in Parkinson's disease. Cell Death Differ.

[167] West AP, Khoury-Hanold W, Staron M, Tal MC, Pineda CM, Lang SM (2015). Mitochondrial DNA stress primes the antiviral innate immune response. Nature.

[168] Moya GE, Rivera PD, Dittenhafer-Reed KE (2021). Evidence for the role of mitochondrial DNA release in the inflammatory response in neurological disorders. Int J Mol Sci.

[169] Inden M, Taira T, Kitamura Y, Yanagida T, Tsuchiya D, Takata K (2006). PARK7 DJ-1 protects against degeneration of nigral dopaminergic neurons in Parkinson's disease rat model. Neurobiol Dis.

[170] Yanagisawa D, Kitamura Y, Inden M, Takata K, Taniguchi T, Morikawa S (2008). DJ-1 protects against neurodegeneration caused by focal cerebral ischemia and reperfusion in rats. J Cereb Blood Flow Metab.

[171] Miyazaki S, Yanagida T, Nunome K, Ishikawa S, Inden M, Kitamura Y (2008). DJ-1-binding compounds prevent oxidative stress-induced cell death and movement defect in Parkinson's disease model rats. J Neurochem.

[172] Yanagida T, Tsushima J, Kitamura Y, Yanagisawa D, Takata K, Shibaike T (2009). Oxidative stress induction of DJ-1 protein in reactive astrocytes scavenges free radicals and reduces cell injury. Oxid Med Cell Longev.

[173] Kitamura Y, Inden M, Kimoto Y, Takata K, Yanagisawa D, Hijioka M (2017). Effects of a DJ-1-binding compound on spatial learning and memory impairment in a mouse model of Alzheimer's disease. J Alzheimers Dis.

[174] Inden M, Yanagisawa D, Hijioka M, Ariga H, Kitamura Y (2017). Therapeutic activities of DJ-1 and its binding compounds against neurodegenerative diseases. Adv Exp Med Biol.

[175] Zhou W, Bercury K, Cummiskey J, Luong N, Lebin J, Freed CR (2011). Phenylbutyrate up-regulates the DJ-1 protein and protects neurons in cell culture and in animal models of Parkinson disease. J Biol Chem.

[176] Boussaad I, Obermaier CD, Hanss Z, Bobbili DR, Bolognin S, Glaab E (2020). A patient-based model of RNA mis-splicing uncovers treatment targets in Parkinson's disease. Sci Transl Med..

[177] Kim RH, Peters M, Jang Y, Shi W, Pintilie M, Fletcher GC (2005). DJ-1, a novel regulator of the tumor suppressor PTEN. Cancer Cell.

[178] Kato I, Maita H, Takahashi-Niki K, Saito Y, Noguchi N, Iguchi-Ariga SM (2013). Oxidized DJ-1 inhibits p53 by sequestering p53 from promoters in a DNA-binding affinity-dependent manner. Mol Cell Biol.

[179] Fan J, Ren H, Jia N, Fei E, Zhou T, Jiang P (2008). DJ-1 decreases Bax expression through repressing p53 transcriptional activity. J Biol Chem.

[180] Bretaud S, Allen C, Ingham PW, Bandmann O (2007). p53-dependent neuronal cell death in a DJ-1-deficient zebrafish model of Parkinson's disease. J Neurochem.

[181] Shinbo Y, Taira T, Niki T, Iguchi-Ariga SM, Ariga H (2005). DJ-1 restores p53 transcription activity inhibited by Topors/p53BP3. Int J Oncol.

[182] Xu J, Zhong N, Wang H, Elias JE, Kim CY, Woldman I (2005). The Parkinson's disease-associated DJ-1 protein is a transcriptional co-activator that protects against neuronal apoptosis. Hum Mol Genet.

[183] Tang B, Xiong H, Sun P, Zhang Y, Wang D, Hu Z (2006). Association of PINK1 and DJ-1 confers digenic inheritance of early-onset Parkinson's disease. Hum Mol Genet.

[184] Moore DJ, Zhang L, Troncoso J, Lee MK, Hattori N, Mizuno Y (2005). Association of DJ-1 and parkin mediated by pathogenic DJ-1 mutations and oxidative stress. Hum Mol Genet.

[185] Heremans IP, Caligiore F, Gerin I, Bury M, Lutz M, Graff J (2022). Parkinson's disease protein PARK7 prevents metabolite and protein damage caused by a glycolytic metabolite. Proc Natl Acad Sci USA.

[186] Krebiehl G, Ruckerbauer S, Burbulla LF, Kieper N, Maurer B, Waak J (2010). Reduced basal autophagy and impaired mitochondrial dynamics due to loss of Parkinson's disease-associated protein DJ-1. PLoS ONE.

[187] Irrcher I, Aleyasin H, Seifert EL, Hewitt SJ, Chhabra S, Phillips M (2010). Loss of the Parkinson's disease-linked gene DJ-1 perturbs mitochondrial dynamics. Hum Mol Genet.

[188] Heo JY, Park JH, Kim SJ, Seo KS, Han JS, Lee SH (2012). DJ-1 null dopaminergic neuronal cells exhibit defects in mitochondrial function and structure: involvement of mitochondrial complex I assembly. PLoS ONE.

[189] Solana-Manrique C, Sanz FJ, Ripolles E, Bano MC, Torres J, Munoz-Soriano V (2020). Enhanced activity of glycolytic enzymes in Drosophila and human cell models of Parkinson's disease based on DJ-1 deficiency. Free Radic Biol Med.

[190] Koide-Yoshida S, Niki T, Ueda M, Himeno S, Taira T, Iguchi-Ariga SM (2007). DJ-1 degrades transthyretin and an inactive form of DJ-1 is secreted in familial amyloidotic polyneuropathy. Int J Mol Med.

[191] Takahashi K, Taira T, Niki T, Seino C, Iguchi-Ariga SM, Ariga H (2001). DJ-1 positively regulates the androgen receptor by impairing the binding of PIASx alpha to the receptor. J Biol Chem.

[192] Taira T, Iguchi-Ariga SM, Ariga H (2004). Co-localization with DJ-1 is essential for the androgen receptor to exert its transcription activity that has been impaired by androgen antagonists. Biol Pharm Bull.

[193] Tillman JE, Yuan J, Gu G, Fazli L, Ghosh R, Flynt AS (2007). DJ-1 binds androgen receptor directly and mediates its activity in hormonally treated prostate cancer cells. Cancer Res.

[194] Goldberg MS, Pisani A, Haburcak M, Vortherms TA, Kitada T, Costa C (2005). Nigrostriatal dopaminergic deficits and hypokinesia caused by inactivation of the familial Parkinsonism-linked gene DJ-1. Neuron.

[195] Niki T, Takahashi-Niki K, Taira T, Iguchi-Ariga SM, Ariga H (2003). DJBP: a novel DJ-1-binding protein, negatively regulates the androgen receptor by recruiting histone deacetylase complex, and DJ-1 antagonizes this inhibition by abrogation of this complex. Mol Cancer Res.

[196] Ishikawa S, Taira T, Takahashi-Niki K, Niki T, Ariga H, Iguchi-Ariga SM (2010). Human DJ-1-specific transcriptional activation of tyrosine hydroxylase gene. J Biol Chem.

[197] Xu CY, Kang WY, Chen YM, Jiang TF, Zhang J, Zhang LN (2017). DJ-1 inhibits alpha-synuclein aggregation by regulating chaperone-mediated autophagy. Front Aging Neurosci.

[198] Zondler L, Miller-Fleming L, Repici M, Goncalves S, Tenreiro S, Rosado-Ramos R (2014). DJ-1 interactions with alpha-synuclein attenuate aggregation and cellular toxicity in models of Parkinson's disease. Cell Death Dis.

[199] Wu J, Xu X, Zheng L, Mo J, Jin X, Bao Y (2021). Nilotinib inhibits microglia-mediated neuroinflammation to protect against dopaminergic neuronal death in Parkinson's disease models. Int Immunopharmacol.

[200] Pagan FL, Wilmarth B, Torres-Yaghi Y, Hebron ML, Mulki S, Ferrante D (2021). Long-term safety and clinical effects of nilotinib in Parkinson's disease. Mov Disord.

[201] Khan A, Johnson R, Wittmer C, Maile M, Tatsukawa K, Wong JL (2021). NPT520-34 improves neuropathology and motor deficits in a transgenic mouse model of Parkinson's disease. Brain.

[202] Lastres-Becker I, Garcia-Yague AJ, Scannevin RH, Casarejos MJ, Kugler S, Rabano A (2016). Repurposing the NRF2 activator dimethyl fumarate as therapy against synucleinopathy in Parkinson's disease. Antioxid Redox Signal.

[203] Jing X, Shi H, Zhang C, Ren M, Han M, Wei X (2015). Dimethyl fumarate attenuates 6-OHDA-induced neurotoxicity in SH-SY5Y cells and in animal model of Parkinson's disease by enhancing Nrf2 activity. Neuroscience.

[204] Kitamura Y, Watanabe S, Taguchi M, Takagi K, Kawata T, Takahashi-Niki K (2011). Neuroprotective effect of a new DJ-1-binding compound against neurodegeneration in Parkinson's disease and stroke model rats. Mol Neurodegener.

[205] Takahashi-Niki K, Inafune A, Michitani N, Hatakeyama Y, Suzuki K, Sasaki M (2015). DJ-1-dependent protective activity of DJ-1-binding compound no. 23 against neuronal cell death in MPTP-treated mouse model of Parkinson's disease. J Pharmacol Sci.

[206] Lev N, Barhum Y, Ben-Zur T, Aharony I, Trifonov L, Regev N (2015). A DJ-1 based peptide attenuates dopaminergic degeneration in mice models of Parkinson's disease via enhancing Nrf2. PLoS ONE.

[207] Post B, van den Heuvel L, van Prooije T, van Ruissen X, van de Warrenburg B, Nonnekes J (2020). Young onset Parkinson's disease: a modern and tailored approach. J Parkinsons Dis.

[208] Postuma RB, Berg D, Stern M, Poewe W, Olanow CW, Oertel W (2015). MDS clinical diagnostic criteria for Parkinson's disease. Mov Disord.

[209] Takeda K, Akira S (2005). Toll-like receptors in innate immunity. Int Immunol.

[210] Park BS, Lee JO (2013). Recognition of lipopolysaccharide pattern by TLR4 complexes. Exp Mol Med.

[211] Gibbons HM, Dragunow M (2006). Microglia induce neural cell death via a proximity-dependent mechanism involving nitric oxide. Brain Res.

[212] Sheppard O, Coleman MP, Durrant CS (2019). Lipopolysaccharide-induced neuroinflammation induces presynaptic disruption through a direct action on brain tissue involving microglia-derived interleukin 1 beta. J Neuroinflammation.

[213] Qin L, Wu X, Block ML, Liu Y, Breese GR, Hong JS (2007). Systemic LPS causes chronic neuroinflammation and progressive neurodegeneration. Glia.

[214] Ling Z, Gayle DA, Ma SY, Lipton JW, Tong CW, Hong JS (2002). In utero bacterial endotoxin exposure causes loss of tyrosine hydroxylase neurons in the postnatal rat midbrain. Mov Disord.

[215] Carvey PM, Chang Q, Lipton JW, Ling Z (2003). Prenatal exposure to the bacteriotoxin lipopolysaccharide leads to long-term losses of dopamine neurons in offspring: a potential, new model of Parkinson's disease. Front Biosci.

[216] Desplats P, Gutierrez AM, Antonelli MC, Frasch MG (2020). Microglial memory of early life stress and inflammation: susceptibility to neurodegeneration in adulthood. Neurosci Biobehav Rev.

[217] Pham TT, Giesert F, Rothig A, Floss T, Kallnik M, Weindl K (2010). DJ-1-deficient mice show less TH-positive neurons in the ventral tegmental area and exhibit non-motoric behavioural impairments. Genes Brain Behav.

[218] Kitada T, Tong Y, Gautier CA, Shen J (2009). Absence of nigral degeneration in aged parkin/DJ-1/PINK1 triple knockout mice. J Neurochem.

[219] Chen L, Cagniard B, Mathews T, Jones S, Koh HC, Ding Y (2005). Age-dependent motor deficits and dopaminergic dysfunction in DJ-1 null mice. J Biol Chem.

[220] Chandran JS, Lin X, Zapata A, Hoke A, Shimoji M, Moore SO (2008). Progressive behavioral deficits in DJ-1-deficient mice are associated with normal nigrostriatal function. Neurobiol Dis.

[221] Yamaguchi H, Shen J (2007). Absence of dopaminergic neuronal degeneration and oxidative damage in aged DJ-1-deficient mice. Mol Neurodegener.

[222] Rousseaux MW, Marcogliese PC, Qu D, Hewitt SJ, Seang S, Kim RH (2012). Progressive dopaminergic cell loss with unilateral-to-bilateral progression in a genetic model of Parkinson disease. Proc Natl Acad Sci USA.

[223] Dave KD, De Silva S, Sheth NP, Ramboz S, Beck MJ, Quang C (2014). Phenotypic characterization of recessive gene knockout rat models of Parkinson's disease. Neurobiol Dis.

[224] Gispert S, Ricciardi F, Kurz A, Azizov M, Hoepken HH, Becker D (2009). Parkinson phenotype in aged PINK1-deficient mice is accompanied by progressive mitochondrial dysfunction in absence of neurodegeneration. PLoS ONE.

